# CGL160-mediated recruitment of the coupling factor CF_1_ is required for efficient thylakoid ATP synthase assembly, photosynthesis, and chloroplast development in Arabidopsis

**DOI:** 10.1093/plcell/koac306

**Published:** 2022-10-17

**Authors:** Bennet Reiter, Lea Rosenhammer, Giada Marino, Stefan Geimer, Dario Leister, Thilo Rühle

**Affiliations:** Plant Molecular Biology Faculty of Biology I, Ludwig-Maximilians-Universität Munich, D-82152 Planegg-Martinsried, Germany; Plant Molecular Biology Faculty of Biology I, Ludwig-Maximilians-Universität Munich, D-82152 Planegg-Martinsried, Germany; Plant Molecular Biology Faculty of Biology I, Ludwig-Maximilians-Universität Munich, D-82152 Planegg-Martinsried, Germany; Zellbiologie/Elektronenmikroskopie NW I/B1, Universität Bayreuth, 95447 Bayreuth, Germany; Plant Molecular Biology Faculty of Biology I, Ludwig-Maximilians-Universität Munich, D-82152 Planegg-Martinsried, Germany; Plant Molecular Biology Faculty of Biology I, Ludwig-Maximilians-Universität Munich, D-82152 Planegg-Martinsried, Germany

## Abstract

Chloroplast ATP synthases consist of a membrane-spanning coupling factor (CF_O_) and a soluble coupling factor (CF_1_). It was previously demonstrated that CONSERVED ONLY IN THE GREEN LINEAGE160 (CGL160) promotes the formation of plant CF_O_ and performs a similar function in the assembly of its c-ring to that of the distantly related bacterial Atp1/UncI protein. Here, we show that in Arabidopsis (*Arabidopsis thaliana*) the N-terminal portion of CGL160 (AtCGL160N) is required for late steps in CF_1_-CF_O_ assembly. In plants that lacked AtCGL160N, CF_1_-CF_O_ content, photosynthesis, and chloroplast development were impaired. Loss of AtCGL160N did not perturb c-ring formation, but led to a 10-fold increase in the numbers of stromal CF_1_ subcomplexes relative to that in the wild type. Co-immunoprecipitation and protein crosslinking assays revealed an association of AtCGL160 with CF_1_ subunits. Yeast two-hybrid assays localized the interaction to a stretch of AtCGL160N that binds to the DELSEED-containing CF_1_-β subdomain. Since Atp1 of Synechocystis (*Synechocystis* sp. PCC 6803) could functionally replace the membrane domain of AtCGL160 in Arabidopsis, we propose that CGL160 evolved from a cyanobacterial ancestor and acquired an additional function in the recruitment of a soluble CF_1_ subcomplex, which is critical for the modulation of CF_1_-CF_O_ activity and photosynthesis.

## Teaser

The green-lineage specific N-terminal domain of CGL160 recruits coupling factor 1 and its lack affects chloroplast development, photosynthesis and thylakoid ATP synthase assembly in Arabidopsis.

IN A NUTSHELL **Background:** Thylakoid ATP synthases are impressive molecular engines that harness the light-driven proton gradient to generate ATP during photosynthesis. Their molecular mode of operation and atomic structure have been elucidated, but their assembly process is still under investigation. Specific auxiliary factors assist in ATP synthase assembly and prevent the accumulation of dead-end products or deleterious intermediates. CGL160 is one such factor and consists of a membrane and an N-terminal domain. The membrane domain of CGL160 is distantly related to bacterial Atp1 proteins, which are also present in cyanobacteria. Previous studies demonstrated that CGL160 promotes efficient formation of the membranous c-ring of thylakoid ATP synthases in *Arabidopsis thaliana*.

**Question:** What is the function of the green lineage-specific N-terminal domain of CGL160 in thylakoid ATP synthase assembly, and what is the evolutionary relationship between CGL160 and Atp1?

**Findings:** Here, we showed that the N-terminal domain of CGL160 is required for the late steps in thylakoid ATP synthase assembly and recruits the stromal ATP synthase intermediate coupling factor CF_1_. The assembly step is critical for chloroplast development in the dark, ATP synthase activity, and photosynthesis in *A. thaliana*. We also revealed that Atp1 from the cyanobacterium *Synechocystis* spec PCC 6803 could functionally replace the membrane domain of CGL160 in *A. thaliana*. These results indicated that Atp1 operates in c-ring assembly in cyanobacteria and that CGL160 evolved from its cyanobacterial ancestor Atp1. However, CGL160 acquired an additional function in linking a soluble ATP synthase intermediate to a membranous subcomplex.

**Next steps:** The next steps are to identify all auxiliary factors required for the assembly of thylakoid ATP synthases and to understand their precise function in ATP synthase formation. Detailed knowledge of the factors and the assembly process could provide elegant strategies for adjusting proton circuits and altering the ATP budget in crops or other photosynthetic organisms.

## Introduction

F-type ATP synthases, which utilize chemiosmotic membrane potentials to generate ATP, are central actors in the energy metabolism of bacteria, mitochondria, and chloroplasts. These biological nanomotors share a largely conserved structure, consisting of a soluble F_1_ and a membrane-bound F_O_ moiety. Bacterial and chloroplast ATP synthases (CF_1_-CF_O_) are closely related with respect to size and subunit composition (Groth and Pohl, 2001; Vollmar et al., 2009; Hahn et al., 2018) and, in contrast to the multimeric mitochondrial ATP synthases, exist as monomers in thylakoid membranes (Daum et al., 2010). In the chloroplasts of land plants, CF_1_-CF_O_ complexes reside exclusively in stroma lamellae and grana-end membranes, because the ∼16-nm stromal extension of CF_1_ prevents its incorporation into the tightly packed grana stacks (Daum et al., 2010).

During photophosphorylation, CF_1_-CF_O_ complexes couple the light-driven generation of the trans-thylakoid proton-motive force (*pmf*) to ADP phosphorylation. The membrane-embedded proteolipid c_14_-ring, together with the non-covalently bound central stalk γε, form the motor unit and drive rotary catalysis by CF_1_. The peripheral stator consists of the subunits a, b, and b′, and is connected to the (αβ)_3_ unit by the δ subunit, which acts as a flexible hinge between CF_1_ and CF_O_ (Murphy et al., 2019). Protons are translocated from the luminal to the stromal side via two aqueous channels in the CF_O_-a subunit. During translocation, each proton enters the access channel and binds to a conserved glutamate residue in subunit CF_O_-c. The c_14_ motor executes an almost complete rotation before releasing the proton into the stroma through the exit channel (Hahn et al., 2018). The counterclockwise rotation of the central stalk in the vicinity of the hexamer triggers alternating nucleotide-binding affinities in the β subunits that ultimately drive ATP generation (reviewed in von Ballmoos et al., 2009; Junge and Nelson, 2015).

As a result of extensive organellar gene transfer during plant evolution, three CF_1_-CF_O_ subunits (b′, γ, δ) are encoded in the nuclear genome, while the remaining CF_1_-CF_O_ genes are organized into two plastid operons. Consequently, two different gene-expression systems must be tightly coordinated with the chloroplast protein import machinery for efficient CF_1_-CF_O_ biogenesis. Several CF_1_-CF_O_ auxiliary factors involved in plastid gene expression have been identified, including proteins involved in mRNA processing (*ATPF* EDITING FACTOR 1, AEF1), mRNA stabilization (PENTATRICOPEPTIDE REPEAT 10, PPR10; BIOGENESIS FACTOR REQUIRED FOR ATP SYNTHASE 2, BFA2), and translation initiation (ATPASE SPECIFIC DEFECT 4, ATP4; TRANSLATION DEFICIENT ATPase 1, TDA1; Pfalz et al., 2009; Eberhard et al., 2011; Zoschke et al., 2012; Yap et al., 2015; Zhang et al., 2019). Moreover, CF_1_-CF_O_ assembly factors ensure correct complex stoichiometry and prevent the accumulation of dead-end products or harmful intermediates that could lead to wasteful ATP hydrolysis or *pmf* dissipation.

As in the case of the bacterial assembly model, plastid CF_1_-CF_O_ complexes are constructed from different intermediates or modules (reviewed in Rühle and Leister, 2015). CF_1_ assembly was first examined using in-vitro reconstitution assays, and was shown to be initiated by α/β dimerization in a chaperone-assisted process (Chen and Jagendorf, 1994). CF_1_ formation depends on CONSERVED IN THE GREEN LINEAGE AND DIATOMS 11 (CGLD11 or alternatively BIOGENESIS FACTORS REQUIRED FOR ATP SYNTHASE 3, BFA3), which is specific to green plants, interacts with the hydrophobic catalytic site of the β-subunit and may prevent aggregation or formation of unfavorable homodimers (Grahl et al., 2016; Zhang et al., 2016). Moreover, PROTEIN IN CHLOROPLAST ATPASE BIOGENESIS (PAB; Mao et al., 2015) and BIOGENESIS FACTORS REQUIRED FOR ATP SYNTHASE 1 (BFA1; Zhang et al., 2018) have been proposed to be required for efficient incorporation of the γ subunit into CF_1_.

Less is known about CF_O_ assembly, and only one accessory factor—CONSERVED ONLY IN THE GREEN LINEAGE160 (CGL160)—has been identified so far (Rühle et al., 2014). Absence of CGL160 in the Arabidopsis (*Arabidopsis thaliana*) mutant *cgl160-1* is associated with a significant reduction (70%–90%) in wild-type (WT) CF_1_-CF_O_ levels, and CF_O_-c subunits accumulate as monomers. Moreover, split-ubiquitin assays have provided evidence that AtCGL160 interacts with CF_O_-c and CF_O_-b. It was therefore concluded that AtCGL160 is required for efficient formation of the c-ring in chloroplasts and shares this function with its distantly related bacterial counterpart ATP SYNTHASE PROTEIN 1 (Atp1 or alternatively named UncI because it is encoded by the first gene in the *unc* operon; Suzuki et al., 2007; Ozaki et al., 2008). Furthermore, AtCGL160 was suggested to participate in CF_1_ assembly into the holo-complex, based on CF_1_ subcomplex co-migration and crosslinking experiments using a putatively specific anti-AtCGL160 antibody (Fristedt et al., 2015).

In this study, the function of the N-terminal domain that is conserved in all CGL160 proteins from the green lineage was investigated in Arabidopsis. The results demonstrate that this domain (AtCGL160N) mediates the critical connection of CF_1_ to CF_O_ assembly modules by interacting with subunit β. Thus, CGL160 emerges as a key auxiliary factor that not only promotes CF_O_ formation but is also involved in late CF_1_-CF_O_ assembly steps.

## Results

### The N-terminal moiety of AtCGL160 is required for efficient photosynthesis and CF_1_-CF_O_ functionality

**Figure 1:**
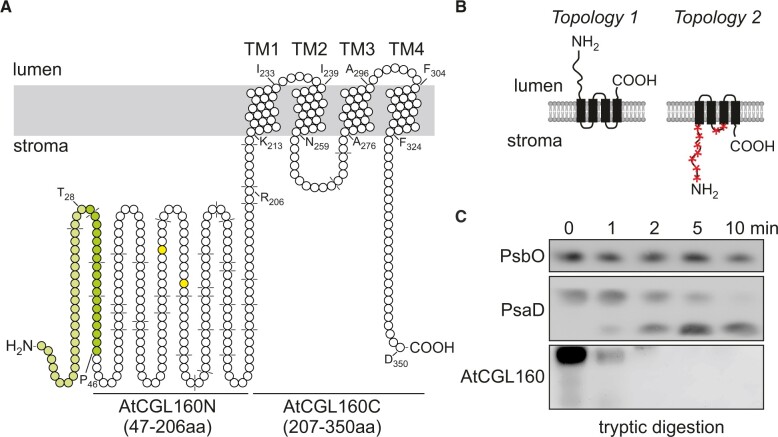
Topology of AtCGL160 and trypsin cleavage-site prediction. A, Transmembrane (TM) domain predictions were obtained from the AtCGL160 UniProt protein accession O82279. Putative trypsin cleavage sites are highlighted in dashed lines and amino acid positions are indicated. The topology was drawn for the full-length sequence of AtCGL160 with Protter (Omasits et al., 2014). The precise length of the AtCGL160 chloroplast TP is unknown. ChloroP chloroplast TP prediction (1–46 aa) and manual annotation (1–28 aa) based on sequence comparison (Supplemental Figure S1) is depicted in green and bright green, respectively. Two conserved serine residues (S111 and S126) are marked in yellow. B, Representation of two putative AtCGL160 topologies. The four TM domains are indicated as black boxes. Accessible trypsin digestion sites are highlighted by red stars. C, Immunoblot of thylakoid membranes of the WT (Col-0) fractionated by SDS-PAGE, untreated (0 min) or treated with trypsin for 1, 2, 5, and 10 min. Blots were probed with antibodies against the lumen-oriented PSII subunit PsbO (antibody AS05 092, Agrisera), the stroma-exposed PSI subunit PsaD (antibody AS09 461, Agrisera) and AtCGL160 (antibody AS12 1853, Agrisera).

CGL160 was identified based on its coregulation with photosynthetic genes in ATTED-II (Obayashi et al., 2009) and its affiliation to the GreenCut suite of proteins (Merchant et al., 2007; Karpowicz et al., 2011). The C-terminal transmembrane segment of CGL160 (∼15 kDa) is distantly related to bacterial Atp1/UncI (Rühle et al., 2014; Fristedt et al., 2015), whereas the larger N-terminal portion of the protein sequence is only conserved in algae, bryophytes, and vascular plants (Supplemental Figure S1). This latter domain of ∼200 amino acids (aa) in Arabidopsis (AtCGL160N) includes a predicted, putative N-terminal chloroplast transit peptide (TP) of 46 aa (Emanuelsson et al., 1999). However, the alignment of AtCGL160 and CGL160N sequences from other species in the green lineage (Supplemental Figure S1) revealed that AtCGL160_29–46aa_ is conserved in vascular plants, indicating incorrect chloroplast TP prediction by ChloroP. Moreover, mass spectrometry has identified several phosphorylated peptides which are derived from positions 106–134 (Reiland et al., 2009; Reiland et al., 2011; Roitinger et al., 2015). Indeed, two conserved putative phosphorylation sites were found in the multiple sequence alignment of CGL160 homologs from species across the green lineage, which correspond to positions S111 and S126 in AtCGL160 (Figure 1, Supplemental Figure S1). Earlier studies have provided experimental evidence for the localization of AtCGL160 to the thylakoid membrane (Rühle et al., 2014; Tomizioli et al., 2014; Fristedt et al., 2015). To gain further insights into the topology of AtCGL160, a protease protection assay was carried out (Figure 1, B and C). In the case of topology 1, all trypsin cleavage sites in AtCGL160 reside in the lumen of the thylakoid and remain fully protected from proteolytic degradation (Figure 1B). Conversely, the stromal orientation of AtCGL160N predicted for topology 2 would expose trypsin cleavage sites and lead to degradation products of less than 2 kD (Figure 1A). To test the accessibility of native AtCGL160N, WT thylakoids were isolated and treated with trypsin for 10 min (Figure 1C). As expected, the luminal PSII subunit PsbO was not affected by the enzyme, whereas the stromally exposed PSI subunit PsaD was susceptible to the protease. AtCGL160N was also efficiently digested, leaving no detectable proteolytic cleavage products, which is consistent with protrusion of the entire N-terminal domain into the stroma, as shown in Topology 2 (Figure 1B).

**Figure 2:**
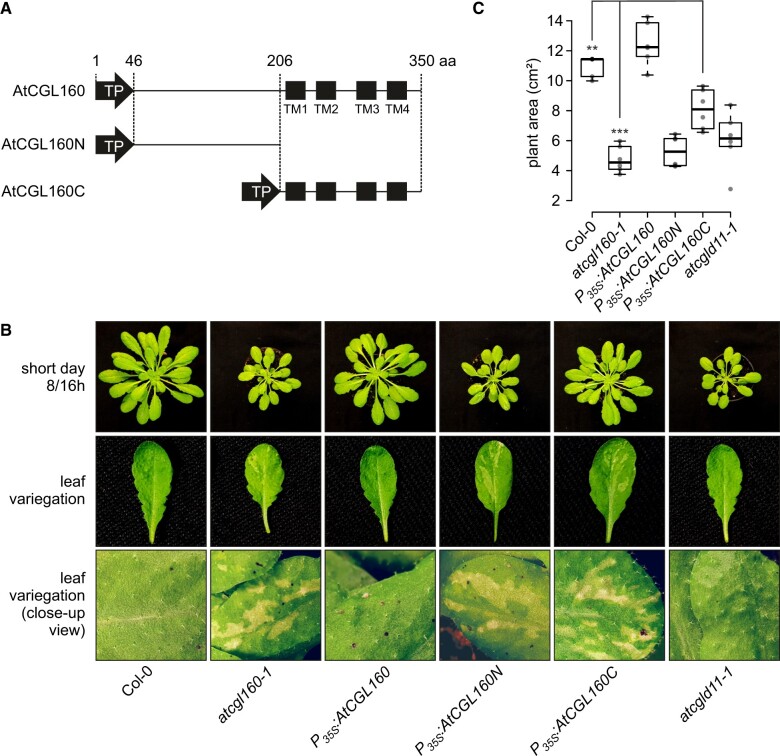
Growth phenotype and leaf variegation of *P_35S_:AtCGL160*, *P_35S_:AtCGL160N*, and *P_35S_:AtCGL160C* plants under short-day conditions. A, Schematic representations of reintroduced AtCGL160 coding sequences. Plants lacking AtCGL160 were transformed with overexpressor constructs harboring the coding sequences for the full-length AtCGL160 (*P_35S_:AtCGL160*) and its N- (*P_35S_:AtCGL160N*) and C-terminal (*P_35S_:AtCGL160C*) segments. Transcription was under the control of the 35S CaMV promoter and targeting to the chloroplast was mediated by the ChloroP-predicted TP of AtCGL160 (TP). Amino acid positions are indicated and predicted TM domains (TM1–TM4) are schematically shown as black boxes. B, Leaf morphology of Col-0, *atcgl160-1*, *P_35S_:AtCGL160, P_35S_:AtCGL160N*, *P_35S_:AtCGL160C*, and *atcgld11-1* plants. C, Leaf areas of six individual plants per genotype were determined 4 weeks after germination. The horizontal lines represent the median, and boxes indicate the 25th and 75th percentiles. Whiskers extend the interquartile range by a factor of 1.5×, and outliers are represented by dots. The effect of the deletion of AtCGL160N in *P_35S_:AtCGL160C* plants on growth under short-day conditions was tested by paired sample *t* test (two-sided). Statistically significant differences are marked with asterisks (*P <* *0.05, **P <* *0.01, and ***P <* *0.001).

To dissect the function of the N-terminal portion of AtCGL160, three different constructs under control of the 35S promoter were cloned, transformed into the *Atcgl160-1* background and screened for complementation (Figure 2, Supplemental Figure S2A). Plants that overexpressed the full-length coding sequence (CDS) of *AtCGL160* served as controls (*P_35S_:AtCGL160*), while the other two genotypes expressed either the CDS of the N-terminal (*P_35S_:AtCGL160N*) or the C-terminal segment (*P_35S_:AtCGL160C*) of the protein (Supplemental Figure S2B). In the case of *P_35S_:AtCGL160C* plants, targeting of the truncated version to chloroplasts was achieved by fusing the CDS of the AtCGL160-derived ChloroP-predicted, putative chloroplast TP (1–46 aa) to that of AtCGL160C (Figure 2A).

Since the commercially available AtCGL160 antibody (Agrisera AS12 1853) displayed non-specific binding to either CF_1_-α or CF_1_-β (Supplemental Figure S3A), an AtCGL160 antibody with no significant cross-reactions to other thylakoid proteins was generated (Supplemental Figure S3B). In the first step of antibody production, the N-terminal part of AtCGL160 conserved in vascular plants (AtCGL160_29–206aa_) was fused to the maltose-binding protein and injected into rabbits. In the second step, antibodies specific for AtCGL160_29–206aa_ were affinity-purified from rabbit antisera using an immobilized fusion protein consisting of AtCGL160_29–206aa_ and glutathione S-transferase. As expected, when the resulting antibody fraction was tested in immunodetection assays, it showed only one distinct signal in the WT sample, which was enriched in extracts of *P_35S_:AtCGL160*, but was absent in the *atcgl160-1* sample (Supplemental Figure S3B).

As was previously demonstrated in complementation analyses with *P_35S_:AtCGL160-eGFP* lines (Rühle et al., 2014), overexpression of the full-length *AtCGL160* rescued the *atcgl160-1* phenotype (Supplemental Figure S2A), as indicated by WT-like growth and restored leaf morphology under short-day conditions (Figure 2, B and C). *P_35S_:AtCGL160N* failed to complement the mutant phenotype (Figure 2, B and C, Supplemental Figure S2A) and AtCGL160N could not be detected in either thylakoid (Supplemental Figure S3B) or leaf extracts (Supplemental Figure S3C). Since *AtCGL160N* transcripts were present in WT-like amounts in *P_35S_:AtCGL160N* plants (Supplemental Figure S2B), the lack of AtCGL160N is probably due to proteolytic degradation owing to its inability to associate correctly with thylakoids. Nevertheless, *P_35S_:AtCGL160N* plants were retained and served as an additional AtCGL160 knockout control. *P_35S_:AtCGL160C* plants with similar overexpression rates to *P_35S_:AtCGL160* plants (Supplemental Figure S2B) were characterized by a significant increase in leaf area compared to the mutant background *atcgl160-1* but were growth-retarded with respect to the WT control. Interestingly, like *atcgl160-1*, *P_35S_:AtCGL160C* plants developed a variegated phenotype in old leaves, which was not found either in the WT or in the CF_1_ assembly mutant *atcgld11-1* (Grahl et al., 2016) under short-day conditions (Figure 2B).

**Figure 3:**
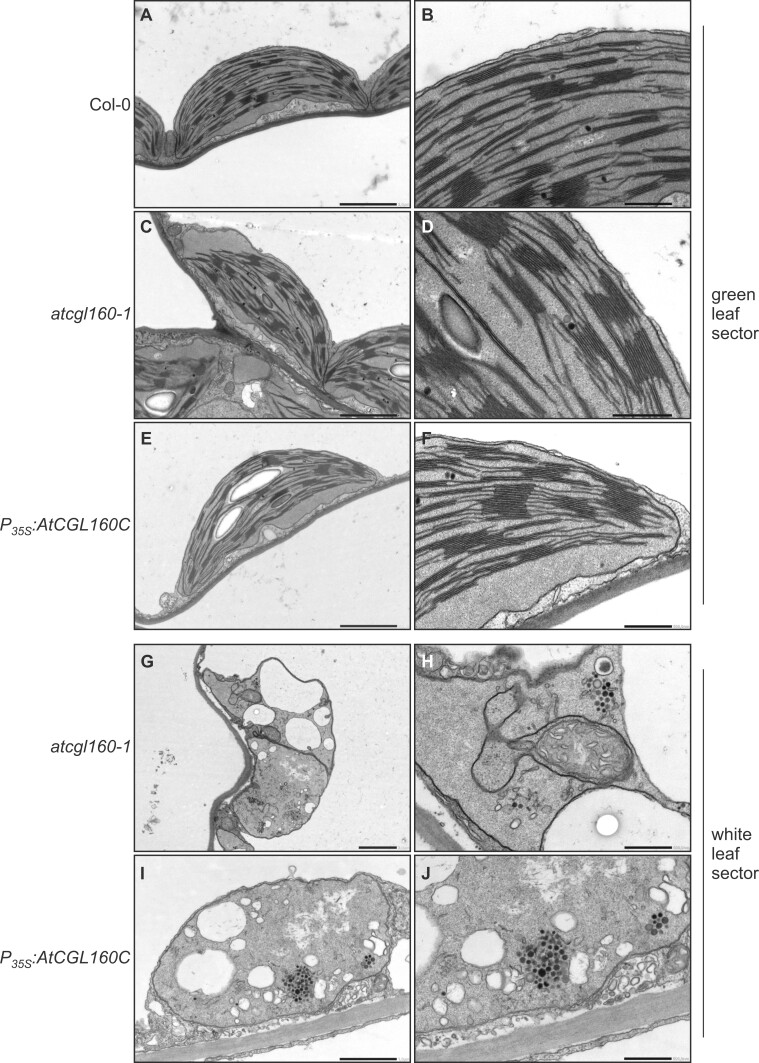
Plastid ultrastructure in white leaf sectors is altered in the absence of AtCGL160N under short-day growth conditions. Electron micrographs of samples from green leaf sections obtained from Col-0 (A and B), *atcgl160-1* (C and D), and *P_35S_:AtCGL160C* (E and F) plants. The ultrastructure of chloroplasts was further examined in samples of white leaf sections obtained from *atcgl160-1* (G and H) and *P_35S_:AtCGL160C* (I and J) plants. The photos on the right show enlargements of the images on the left. The scale bar corresponds to 2 µm in (A, C, E, and G), 1 µm in (I) and 0.5 µm in (B, D, F, H, and J).

To analyze the leaf phenotype in more detail, we carried out electron microscopic analyses of Col-0, *atcgl160-1* and *P_35S_:AtCGL160C* plants (Figure 3). In these genotypes, the chloroplast ultrastructure in preparations from green leaf sections was unchanged with regard to grana number per chloroplast and grana height distribution (Figure 3, A–F, Supplemental Figure S4). These observations in *atcgl160-1*, together with previous ultrastructural analyses of the CF_1_ assembly mutant line *atcgld11-1* (Grahl et al., 2016) and spinach (*Spinacia oleracea*) chloroplasts (Daum et al., 2010), support the idea that CF_1_-CF_O_ complexes are not physically involved in thylakoid curvature formation. Examination of white leaf sections in *atcgl160-1* and *P_35S_:AtCGL160C* revealed the absence of thylakoids in plastids, accompanied by the appearance of plastoglobuli in densely packed stromal clusters (Figure 3, G–J). Furthermore, large vesicles were observed, which also point to increased catabolic activity and degradation processes in *atcgl160-1* and *P_35S_:AtCGL160C* plastids. Another finding was the inclusion of mitochondria in degraded plastids, which was also observed, to a lesser extent, in white leaf sectors of *P_35S_:AtCGL160C*.

**Figure 4:**
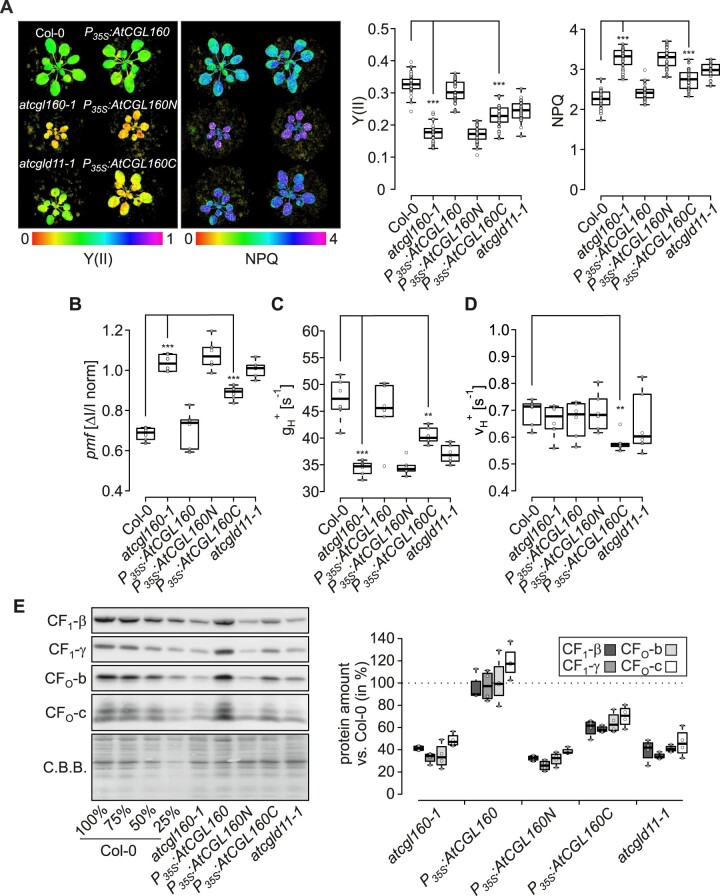
Lack of AtCGL160N perturbs photosynthesis and CF_1_-CF_O_ integrity. A, PSII quantum yield (Y(II)) and heat dissipation (NPQ) of Col-0, *atcgl160-1*, *P_35S_:AtCGL160, P_35S_:AtCGL160N*, *P_35S_:AtCGL160C*, and *atcgld11-1* plants were determined using an Imaging-PAM system (Walz) and are displayed on a rainbow color scale (left panel; 0–1 for Y(II) and 0–4 for NPQ). Y(II) (middle panel) and NPQ (right panel) analyses from six plants per genotype and five leaves (*n* = 30) were analyzed at 185 µmol photons m^−2^ s^−1^. B, DIRK derived from ECS signals were recorded after 10 min of illumination from six individual plants grown under short-day conditions. To determine the *pmf*, total amplitude of the P515 differential absorption signal was normalized to a single turnover flash 4 min after the ECS measurement. C, Proton conductivity of the thylakoid membrane was determined from ECS signal relaxation rates, which were fitted to a first-order decay function. The inverse of the calculated rate constant was expressed as $g_{H}^{+}$ [s^−1^]. Measurements were obtained from six individual plants grown under short-day conditions. D, The proton flux parameter ν_H_^+^ was determined from the initial rate of decay of the ECS signal. Measurements were conducted on six individual plants grown under short-day conditions. E, Steady-state levels of immunodetected CF_1_-CF_O_ marker subunits. After fractionation of thylakoid proteins on SDS-PAGE and transfer to PVDF membranes, blots were probed with antibodies against CF_1_-β, CF_1_-γ, CF_O_-b, and CF_O_-c. Coomassie Brilliant Blue (C.B.B.) staining is shown as loading control. For quantification, signals from four technical replicates of each marker subunit were normalized to signals detected in Col-0 samples. Horizontal lines represent the median, and boxes indicate the 25th and 75th percentiles. Whiskers extend the interquartile range by 1.5×. The effect of the deletion of AtCGL160N on photosynthetic parameters of *P_35S_:AtCGL160C* plants shown in panels (A–D) was tested in paired-sample *t* tests (two-sided). Statistically significant differences are marked with asterisks (*P <* *0.05, **P <* *0.01, and ***P <* *0.001).

To test whether disruption of AtCGL160N impairs photosynthesis and CF_1_-CF_O_ activity, measurements of chlorophyll *a* fluorescence and electrochromic shift (ECS) were carried out on Col-0, *atcgl160-1*, *P_35S_:AtCGL160, P_35S_:AtCGL160N*, *P_35S_:AtCGL160C*, and *atcgld11-1* plants (Figure 4, A–D). As expected, the CF_1_-CF_O_ assembly mutants *atcgl160-1* and *atcgld11-1* showed lower effective PSII quantum yields [Y(II)], higher heat dissipation (indicated as non-photochemical quenching, NPQ) and increased *pmf*, but lower proton conductivity ($g_{H}^{+}$) through the thylakoid membrane compared to the WT control. *P_35S_:AtCGL160* and *P_35S_:AtCGL160N* plants displayed similar levels of NPQ, *pmf* and $g_{H}^{+}$ to the WT and the CF_1_-CF_O_ assembly mutant *atcgld11-1*, respectively. Notably, photosynthetic parameters in the *P_35S_:AtCGL160C* line were only partially restored and Y(II) (Figure 4A), as well as proton flux (ν_H_^+^) through the photosynthetic apparatus were decreased compared to that in Col-0 (Figure 4D).

To assess the integrity of the CF_1_-CF_O_ complex in thylakoids, marker subunits were immunodetected in *atcgl160-1*, *P_35S_:AtCGL160, P_35S_:AtCGL160N*, *P_35S_:AtCGL160C*, and *atcgld11-1* plants, and quantified relative to Col-0 samples (Figure 4E). Levels of CF_1_-β, CF_1_-γ, CF_O_-b and CF_O_-c were restored to normal in *P_35S_:AtCGL160* but reduced to about 60%–65% of WT amounts in *P_35S_:AtCGL160C* plants. Transformation with the *P_35S_:AtCGL160N* construct had no effect on CF_1_-CF_O_ subunit levels in the *atcgl160-1* mutant.

Overall, overexpression of the Atp1/Unc1-like AtCGL160 domain alone (AtCGL160C) in the *atcgl160-1* background only partially restored CF_1_-CF_O_ amounts (Figure 4E) and activity (Figure 4C). Consequently, photosynthesis was downregulated and growth of *P_35S_:AtCGL160C* plants was impaired (Figure 2C), which could be attributed to more highly activated ΔpH-dependent quenching mechanisms (Figure 4A). We deduced from these results that AtCGL160N might also be involved in CF_1_-CF_O_ assembly at steps other than CF_O_-c ring formation.

### Stromal accumulation of CF_1_ in the absence of AtCGL160N

**Figure 5:**
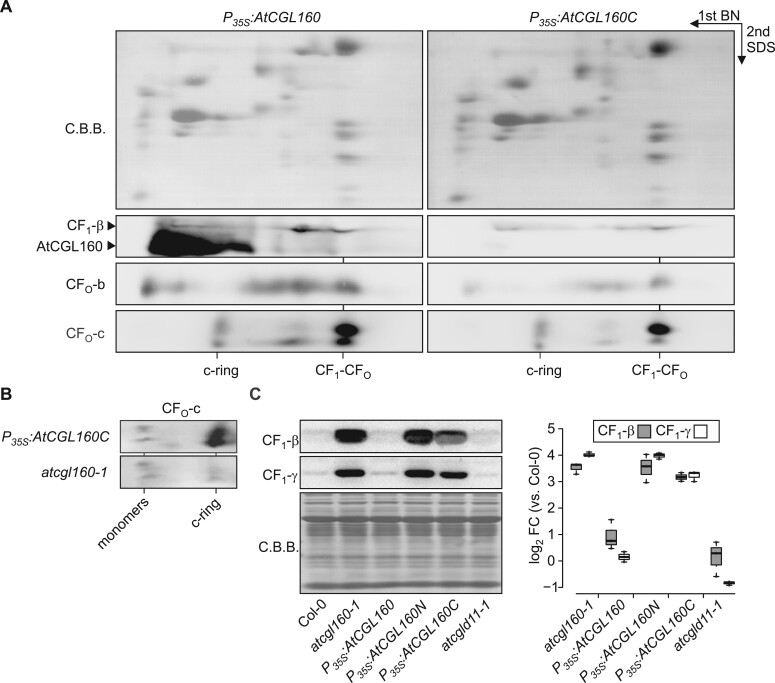
CF_1_-CF_O_ assembly is perturbed in the absence of AtCGL160N. A, Thylakoid complexes from *P_35S_:AtCGL160* and *P_35S_:AtCGL160C* plants were solubilized with *n*-dodecyl *β*-D-maltoside (1% [w/v]) and further separated by Blue-Native (BN, first dimension) and SDS-PAGE (SDS, second dimension). After protein transfer, PVDF membranes were probed with antibodies against CF_1_-β, CF_O_-b, and CF_O_-c, and CF_1_-β blots were subsequently exposed to anti-AtCGL160 antibodies (generated this study). Positions of the ATP synthase holo-complex (CF_1_-CF_O_) and the c-ring are indicated. Coomassie Brilliant Blue G-250 (C.B.B.) staining of PVDF membranes is shown as loading control. B, C-ring assembly in *atcgl160-1* and *P_35S_:AtCGL160C* plants. Increased amounts of thylakoid complexes (corresponding to 120 µg total chlorophyll) were solubilized and fractionated by BN/SDS-PAGE. Blots were probed with an antibody against CF_O_-c. Positions of free c-monomers and the assembled c-ring are indicated. C, CF_1_-β and CF_1_-γ enrichment in stromal extract, which was isolated from Col-0, *atcgl160-1*, *P_35S_:AtCGL160, P_35S_:AtCGL160N*, *P_35S_:AtCGL160C*, and *atcgld11-1* rosette leaves. Signals of three CF_1_-β and CF_1_-γ immunodetection assays were quantified and are shown on a logarithmic scale. Horizontal lines represent the median, boxes indicate the 25th and 75th percentiles and whiskers extend the interquartile range by a factor of 1.5×.

To investigate the effects of deletion of AtCGL160N on CF_1_-CF_O_ assembly, we performed BN/SDS-PAGE (2D-PAGE) analysis on thylakoids isolated from *P_35S_:AtCGL160* and *P_35S_:AtCGL160C* plants grown under short-day conditions. Consistent with the accumulation of CF_1_-CF_O_ marker subunits in Figure 4E, CF_1_-β, CF_O_-b, and CF_O_-c levels were reduced in *P_35S_:AtCGL160C* compared to the levels in plants that overexpressed the full-length CDS of AtCGL160 (Figure 5A). No accumulation of pre-complexes was observed, as amounts of free proteins, and components of the c-ring, CF_1_, and the holo-complex were reduced uniformly. To assess the assembly status of the c-ring in more detail, we carried out 2D-PAGE with increased amounts of *atcgl160-1* and *P_35S_:AtCGL160C* thylakoids (Figure 5B). C-ring levels were considerably higher in *P_35S_:AtCGL160C* than in the *atcgl160-1* mutant background. We also examined CF_1_ accumulation in the stroma of Col-0, *P_35S_:AtCGL160, P_35S_:AtCGL160N*, *P_35S_:AtCGL160C*, and *atcgld11-1* plants (Figure 5C), since CF_1_-CF_O_ assembly takes place in a modular fashion and involves distinct thylakoid-integral and soluble intermediates. Strikingly, CF_1_-β and CF_1_-γ were enriched about 10-fold in the stroma of *atcgl160-1*, *P_35S_:AtCGL160N*, and *P_35S_:AtCGL160C*, but were detected in close to WT levels in *P_35S_:AtCGL160* and *atcgld11-1* plants. In-depth 2D-PAGE analysis of CF_1_ intermediates in *atcgl160-1*, and comparison with results from the co-migration database for photosynthetic organisms (PCom-DB, Takabayashi et al., 2017), revealed that in *atcgl160-1* stromal CF_1_-β and CF_1_-γ were predominantly present in an α_3_β_3_γε complex that lacked subunit CF_1_-δ (Supplemental Figure S5).

We concluded that reintroduction of the transmembrane Atp1/Unc1-like domain of AtCGL160 restores c-ring formation, but leads to an overall reduction in CF_1_-CF_O_ levels due to a defect in the attachment of CF_1_ to a membrane-integral CF_O_ intermediate.

### AtCGL160 interacts physically with CF_1_-containing complexes

**Figure 6:**
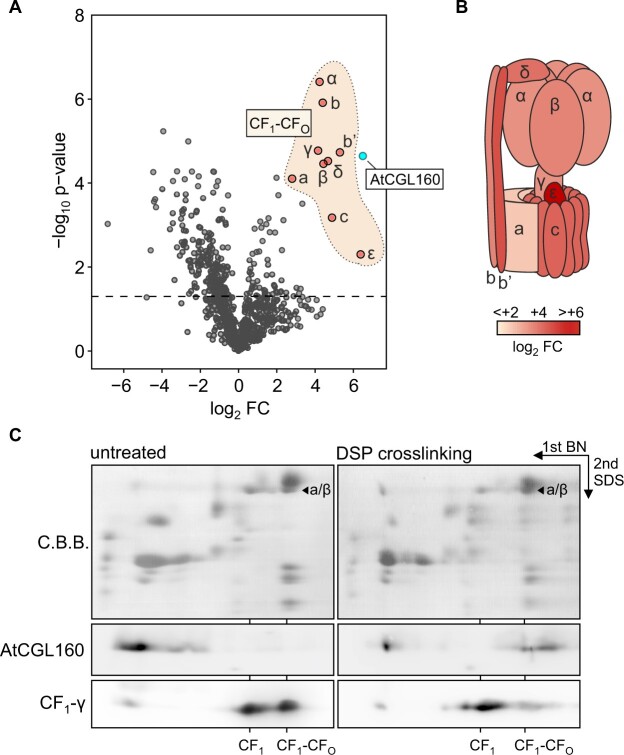
AtCGL160 association with CF_1_ subunits. A, Co-immunoprecipitation analyses with the newly generated antibody were carried out with solubilized thylakoids isolated from *P_35S_:AtCGL160*, while *P_35S_:AtCGL160C* plants served as the negative control. Co-immunoprecipitated proteins were further subjected to tryptic digestion, and peptides were analyzed by liquid chromatography coupled to mass spectrometry. Data for differentially enriched proteins are presented in a volcano plot. Each point indicates a different protein, ranked according to *P*-value (*y*-axis, −log_10_ of *P*-values) and relative abundance ratio (*x*-axis, log_2_ Fold Change *P_35S_:AtCGL160*/*P_35S_:AtCGL160C*). Protein candidates of interest are labelled in red. The dashed line indicates a negative log_10_*P*-value of 1.5. Blue and red dots highlight AtCGL160 and CF_1_-CF_O_ subunits, respectively. B, Schematic representation of differentially enriched subunits in a CF_1_-CF_O_ cartoon. Relative amounts of co-immunoprecipitated CF_1_-CF_O_ subunits are shown in colors on a log_2_ FC scale from white (log_2_ FC < 2) to red (log_2_ FC > 6). Co-immunoprecipitation assays were carried out with three independent biological replicates. In each replicate, co-immunoprecipitation was performed with solubilized thylakoids isolated from ∼10 g leaf fresh weight of *P_35S_:AtCGL160* or *P_35S_:AtCGL160C* plants. C, Co-migration of AtCGL160 with CF_1_-CF_O_ in crosslinking experiments. Two-dimensional BN/SDS-PAGE analysis was used to compare untreated thylakoid extracts of the WT (Col-0) with extracts that had been crosslinked with DSP. Blots of the second dimension were probed with antibodies against AtCGL160 and CF_O_-γ. The positions of CF_1_-CF_O_, the CF_1_ intermediate, and the free protein fraction are indicated based on the mobility of α/β on the C.B.B. stained gel.

To pinpoint the role of AtCGL160 in the recruitment of CF_1_ to a membrane-integral CF_O_ intermediate, protein interactions were assessed in co-immunoprecipitation (co-IP) assays (Figure 6, A and B). NP40-solubilized thylakoid proteins from *P_35S_:AtCGL160* plants grown under short-day conditions were chosen as co-IP input and pulled-down protein amounts were compared to those recovered in co-IP experiments carried out on thylakoid protein extracts of *P_35S_:AtCGL160C*. Plants devoid of AtCGL160 were not considered for use as negative controls, since the reduction in CF_1_-CF_O_ levels observed in *atcgl160-1* (and *P_35S_:AtCGL160N*; Figure 4E) might lead to misinterpretation of differential co-IP experiments. Quantitative data for precipitated proteins were obtained by tryptic digestion and subsequent peptide-fragment analysis using liquid chromatography coupled to mass spectrometry (Figure 6A). As expected, AtCGL160 was pulled down efficiently from *P_35S_:AtCGL160* extracts (log_2_ FC ∼6.5). Moreover, all CF_1_-CF_O_ subunits were identified in co-IPs (Figure 6, A and B) with high differential enrichment levels for the subunits α, β, γ, δ, ε, b, b′, and c (log_2_ FC > 4.4). Subunit CF_O_-a was co-immunoprecipitated at lower levels (log_2_ FC ∼2.8). The pull-down of CF_1_ subunits was confirmed by immunodetection assays of the two marker subunits CF_1_-β and CF_1_-γ, which were only detectable in co-IP output fractions obtained from *P_35S_:AtCGL160* samples (Supplemental Figure S6). Other known CF_1_-CF_O_ assembly factors were not co-immunoprecipitated (Supplemental Table S1), indicating that AtCGL160 is associated with a late CF_1_-CF_O_ assembly stage or the fully assembled complex from which other auxiliary factors had already dissociated.

Since ALBINO 4 (ALB4), which is a member of the bacterial ALBINO 3 (ALB3)/OXIDASE ASSEMBLY 1 (Oxa1)/YidC protein insertase family, was previously proposed to participate in the linkage of a CF_1_ to a CF_O_ assembly module (Benz et al., 2009), but was not pulled down in co-IP assays (Supplemental Table S1), the amount of thylakoid-associated CF_1_ complexes was re-assessed in *atalb4-1* (SALK_136199C) mutants and compared to levels identified in *atcgl160-1* plants (Supplemental Figure S7). Thylakoids were isolated from Col-0, *atcgl160-1* and *atalb4-1* plants grown under short-day conditions and subjected to immunodetection assays of subunit CF_1_-β. CF_1_-β levels bound to *atcgl160-1* thylakoids were reduced to 23 ± 13% of the WT level, while no deviation to the WT control could be observed for thylakoid-associated CF_1_-β content in *atalb4-1* samples (106 ± 30%).

To confirm the association of AtCGL160 with CF_1_-containing complexes, crosslinking experiments were also carried out (Figure 6C). To this end, thylakoid membranes of WT plants were treated with the crosslinker dithiobis(succinimidyl propionate) (DSP), and subsequently subjected to 2D-PAGE and immunodetection of AtCGL160 and CF_1_-CF_O_ marker subunits. In analyses with untreated thylakoid samples, AtCGL160 migrated predominantly in the monomer fraction. After crosslinking, AtCGL160 could be detected at a molecular mass range that corresponded to that of the CF_1_-CF_O_ holo-complex.

In summary, co-IP of all CF_1_-CF_O_ subunits with an AtCGL160-specific antibody, together with the observation that AtCGL160 co-migrated with the CF_1_-CF_O_ holo-complex after DSP cross-linking, corroborates the involvement of AtCGL160 in the functional integration of CF_1_ into the holo-complex at a late step in CF_1_-CF_O_ assembly.

### AtCGL160N interacts with CF_1_-β in yeast two-hybrid assays

**Figure 7:**
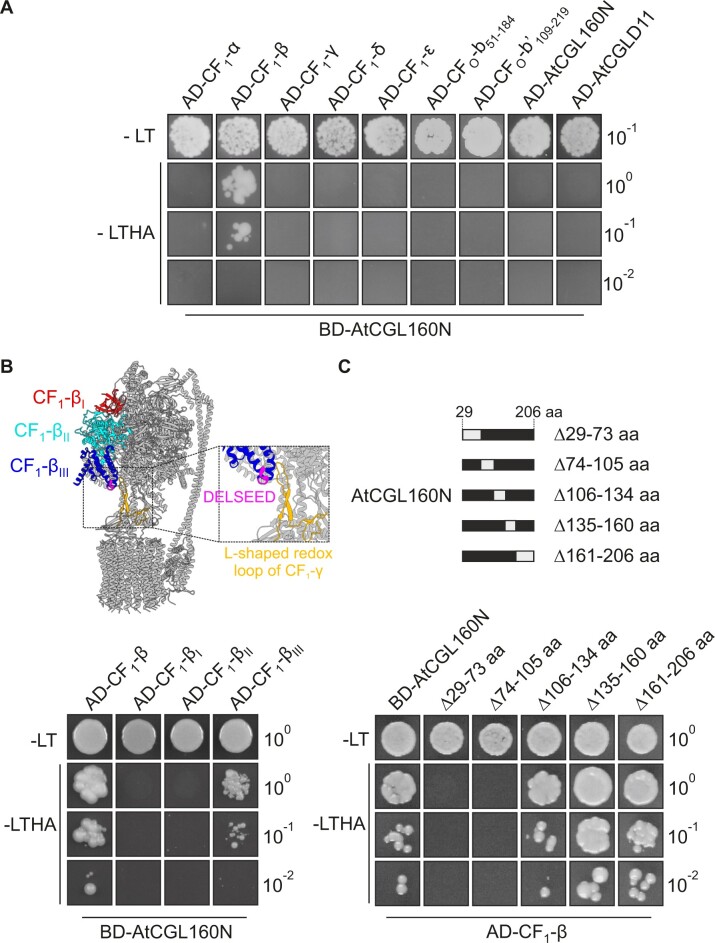
AtCGL160N interaction studies in yeast two-hybrid assays. A, Interactions of AtCGL160 with CF_1_-CF_O_ structural components exposed on the stromal side of thylakoids were tested by transformation of a construct that fuses AtCGL160N to the GAL4 DNA-BD (BD-AtCGL160). Cells were then co-transformed with constructs coding for GAL4 AD fused to CF_1_-α, β, γ, δ, ε, CF_O_-b_51–184_ or CF_O_-b′_109–219_, as well as AtCGL160N or AtCGLD11. B, Interaction of AtCGL160N with structural domains of CF_1_-β. Yeast cells carrying a construct coding for BD-AtCGL160 were transformed with constructs coding for AD-CF_1_-β_I_, AD-CF_1_-β_II_, and AD-CF_1_-β_III_. Structural domains of the CF_1_-β are colored in red (Domain I), turquoise (Domain II), and blue (Domain III). The conserved DELSEED motif is shown in purple, and the L-shaped redox loop of CF_1_-γ in orange. The atomic model of CF_1_-CF_O_ was obtained from the PDB database (ID: 6fkh, Hahn et al., 2018) and formatted with ChimeraX (Pettersen et al., 2021). C, Mapping of the AtCGL160N interaction site. Consecutive regions (grey boxes) coding for segments of the soluble AtCGL160 domain were omitted from the BD-AtCGL160N vector and co-transformed with AD-CF_1_-β into competent yeast cells. Transformations in all assays were verified by plating on permissive medium lacking Leu and Trp (-LT). Interactions were then tested on selective medium (-Leu/-Trp/-His/-Ade, [-LTHA]) by plating equal numbers of yeast cells in serial dilutions (10°, 10^−1^, and 10^−2^). All experiments were performed at least twice in independent co-transformation assays. Note that the reciprocal constructs BD-CF_1_-β and AD-AtCGL160N did not interact in yeast two-hybrid assays (Supplemental Figure S8).

Interactions between the stroma-oriented AtCGL160N domain and individual CF_1_ subunits were further examined by yeast two-hybrid experiments (Figure 7). Since AtCGL160_29–46aa_ is conserved in other vascular plants (Supplemental Figure S1) and might be part of the mature protein, it was considered in yeast two-hybrid assays. To this end, a construct coding for a fusion of AtCGL160N_29–206aa_ to the GAL4-binding domain (BD) was co-transformed into yeast cells together with constructs coding for GAL4 activation domain (AD) fusions to all CF_1_ subunits (α, β, γ, δ, ε). Moreover, the BD-AtCGL160N interaction was tested with AD fusions to the soluble parts of the stator subunits b and b′, AtCGL160N, and CF_1_ assembly factor AtCGLD11. As a result, only yeast cells carrying constructs for AD-CF_1_-β and BD-AtCGL160N could grow on selective medium (Figure 7A). However, the reciprocal constructs BD-CF_1_-β and AD-AtCGL160N did not interact (Supplemental Figure S8). To narrow down the CF_1_-β interaction site, additional AD fusion constructs were cloned that encoded three different CF_1_-β subdomains (Figure 7B) defined in earlier studies (Groth and Pohl, 2001; Zhang et al., 2016). Domain I comprises a thylakoid-distal β-barrel structure and interacts with CF_1_-δ. Domain II harbors the catalytic site involved in ATP generation or hydrolysis. The thylakoid-proximal domain III contains the conserved “DELSEED” motif, which is required for CF_1_-γ/ε-dependent regulation of ATP hydrolysis and synthase activity (Kanazawa et al., 2017; Hahn et al., 2018). When tested on restrictive medium, only cells harboring AD-CF_1_-β_III_ together with BD-AtCGL160N could grow. In a reciprocal approach, coding sequences of AtCGL160N were deleted successively from the BD-AtCGL160N construct (Δ29–73, Δ74–105, Δ106–134, Δ135–160, and Δ161–206 aa) and tested for AD-CF_1_-β interaction in yeast cells (Figure 7C). Only the Δ29–73 and Δ74–105 aa deletions resulted in an absence of growth, while yeast strains with deletion constructs of Δ106–134, Δ135–160, and Δ161–206 aa were able to proliferate on selective medium (Figure 7C). Thus, the interaction between AtCGL160 and CF_1_ involves AtCGL160_29–105_ and the thylakoid-proximal domain of CF_1_-β_III_, while the phosphorylation hotspot identified in the protein segment 106–134 aa (Figure 1A) is dispensable for the interaction.

### Atp1 of Synechocystis (*Synechocystis* sp. PCC 6803) can functionally replace the membrane domain of AtCGL160 in Arabidopsis

The membrane domain of AtCGL160 shares moderate sequence similarity to Atp1 (SynAtp1) of Synechocystis and is encoded by the first gene in the *atp1* operon (Supplemental Figure S9A). To investigate its function in cyanobacteria, a knockout of the *SynAtp1* gene was generated. Since keeping the endogenous promoter in the homologous recombination cassette would result in unwanted recombination events without deletion of *synatp1*, it was replaced by the strong *psbA2* promoter *P_psbA2_* (Supplemental Figure S9A). As a reference, a Synechocystis strain (*P_psbA2_:synatp1*) was generated in which the intact *synatp1* operon was under control of *P_psbA2_*. Full segregation could be achieved for control strain *P_psbA2_:synatp1* but not for *P_psbA2_:Δsynatp1* even in the presence of 500 µg mL^−1^ kanamycin (Supplemental Figure S9B). Reverse transcription-quantitative PCR (RT-qPCR) analyses revealed that *synatp1* expression in *P_psbA2_:synatp1* and *P_psbA2_:Δsynatp1* was increased ∼4-fold and decreased to ∼10%, respectively, compared with expression levels of the WT control (Supplemental Figure S9C). Growth analyses (Supplemental Figures S9D and S10A) gave rise to similar maximal doubling times for the WT and the *P_psbA2_:synatp1* control strain under mixotrophic (∼9 h) and autotrophic (∼23 h) growth conditions. However, maximal doubling times of the non-segregated *P_psbA2_:Δsynatp1* were slightly longer under mixotrophic (∼10 h) and more pronounced under autotrophic (∼31 h) conditions (Supplemental Figure S10A). The effect of promoter replacement and SynAtp1 disruption was further investigated by gas exchange measurements using a Clark-type oxygen electrode (Supplemental Figure S10B). Neither oxygen consumption in the dark nor production rates at 400 µmol photons m^−2^ s^−1^ were altered in both strains compared to that in the WT control. Investigations of CF_1_-CF_O_ marker subunit and CF_O_-c multimer accumulation by immunodetection showed no apparent differences among the genotypes (Supplemental Figure S10C). We concluded from those results that the endogenous promoter of the *synatp1* operon could be replaced by the strong *psbA2* promoter and *synatp1* overexpression did not affect respiration, photosynthesis or CF_1_-CF_O_ accumulation. Moreover, SynAtp1 might have an essential function in Synechocystis, since segregation could not be achieved even in the presence of high selection pressure. As a consequence, residual genomic copies carrying the intact *synatp1* gene under control of the endogenous *synatp1* promoter and *synatp1* expression levels of ∼10% were still sufficient in the non-segregated *P_psbA2_:Δsynatp1* strain to maintain CF_1_-CF_O_ assembly at WT levels.

**Figure 8:**
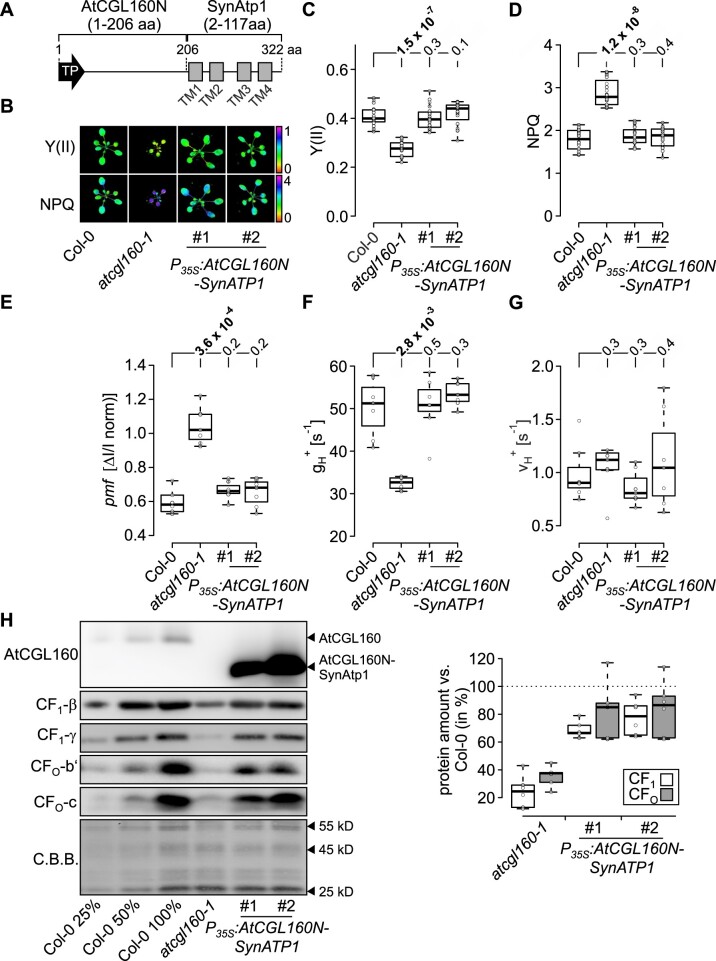
Synechocystis Atp1 (SynAtp1) can functionally substitute for the membrane domain of AtCGL160 in Arabidopsis. A, Replacement of the AtCGL160 membrane domain by SynAtp1. TP, TM domains of SynAtp1 (TM1–TM4) and amino acid positions are indicated. B, Imaging-PAM analysis of two independent *P_35S_:AtCGL160N-SynATP1* overexpressor plants (*atcgl160-1* background) grown under short-day conditions. Effective quantum yield of photosystem II (Y(II)) and NPQ were recorded 600 s after light induction (110 µmol photons m^−2^ s^−1^) and are displayed on a false color scale from 0 to 1 and 0 to 4, respectively. C, Y(II) analyses of *P_35S_:AtCGL160N-SynATP1* overexpressor plants. Y(II) was quantified from five plants per genotype and four measurements taken from individual leaves per plant (*n* = 20). D, NPQ analyses of *P_35S_:AtCGL160N-SynATP1* overexpressor plants. NPQ was quantified from five plants per genotype with four measurements taken from individual leaves per plant (*n* = 20). E, The *pmf* determined in ECS measurements. F, Thylakoid membrane proton conductivity $g_{H}^{+}$ [s^−1^] derived from fast ECS kinetics analyses. G, Proton flux ν_H_^+^ through the photosynthetic apparatus. ECS parameters (E–G) were analyzed on single leaves of 6–7 individual plants per genotype. For statistical analyses (C–G), the non-parametric Kruskal–Wallis test was performed, followed by pairwise Dunn’s tests. The *P*-values were adjusted on an experiment level using the Benjamini–Hochberg method. *P*-values (*P* ≤ 0.05) are displayed in bold. H, Immunodetection assays of AtCGL160N and CF_1_-CF_O_ marker subunits in thylakoids isolated from two *P_35S_:AtCGL160N-SynATP1* overexpressor lines #1 and #2. The newly generated antibody was employed to detect the fusion protein. Coomassie brilliant blue G-250 staining (C.B.B.) of a PVDF membrane is provided as loading control. Thylakoid-associated CF_1_ (CF_1_-β and CF_1_-γ) and CF_O_ (CF_O_-b′ and CF_O_-c) subunits were quantified from three biological replicates (3–6 pooled plants per replicate) and amounts (in %) were referred to WT samples. Horizontal lines in boxplots shown in C and E represent the median, boxes indicate the 25th and 75th percentiles and whiskers extend the interquartile range by a factor of 1.5×.

Since functional analyses with the non-segregated *P_psbA2_:Δsynatp1* Synechocystis strain turned out to be challenging, we pursued a strategy of cross-species complementation and replaced the membrane domain of AtCGL160 with SynAtp1 (Figure 8). Accordingly, an overexpressor construct coding for the fusion of AtCGL160N (1–206 aa) and SynAtp1 (2–117 aa) was transformed into the *atcgl160-1* background (Figure 8A). Two independent *P_35S_:AtCGL160N-SynATP1* progenies were examined with respect to photosynthesis and chloroplast ATP synthase activity (Figure 8, B–G). Remarkably, both lines restored the photosynthetic phenotype to normal, as Y(II) and NPQ levels determined in light induction experiments were comparable to those of the WT control (Figure 8, B–D). The same observation could be made for the *pmf* and proton conductivity, which were recovered to WT levels in the two *P_35S_:AtCGL160N-SynATP1* lines (Figure 8, E and F). No effect on proton flux could be identified in *P_35S_:AtCGL160N-SynATP1* overexpressor plants (Figure 8G). Accumulation and thylakoid localization of AtCGL160N-SynAtp1 in the *atcgl160-1* background could be confirmed by immunodetection using the AtCGL160N-specific antibody (Figure 8H). For both lines, the presence of AtCGL160N-SynAtp1 led to a partial restoration of thylakoid-associated CF_1_ (70%–80%) and CF_O_ (80%–90%) to WT levels (Figure 8H).

In summary, SynAtp1 could replace AtCGL160C and function in the chloroplast CF_1_-CF_O_ assembly process. However, a very high level of the chimeric AtCGL160N-SynAtp1 protein was required to achieve partial restoration in CF_1_-CF_O_ content in Arabidopsis. Conversely, a conserved role in CF_O_ assembly could be inferred for Atp1 in Synechocystis, as AtCGL160C alone was able to promote c-ring formation (Figure 5B).

## Discussion

### AtCGL160 evolved from the cyanobacterial ancestor Atp1

The assembly of protein complexes generally depends on a network of auxiliary factors that must exhibit a high degree of compatibility with their substrates. For instance, functional Rubisco was assembled in *Escherichia coli* only in the presence of the chloroplast chaperonin system CHAPERONIN 60 (Cpn60)/CHAPERONIN 20 (Cpn20), which could not be replaced by the bacterial GroEL (large protein encoded in the *GroE* operon)/GroES (small protein encoded in the *GroE* operon) system (Aigner et al., 2017). It is therefore particularly noteworthy that SynAtp1 was compatible with the CF_O_ assembly process in chloroplasts despite having only moderate sequence similarity to the membrane domain of AtCGL160 (Rühle et al., 2014). However, CF_O_-c sequence conservation from cyanobacteria to plants with 80% identical amino acids is high (Lill and Nelson, 1991), and might account for functional cross-species complementation with a construct encoding an AtCGL160N-SynAtp1 fusion (Figure 8). From these results, we concluded that CGL160 evolved from the cyanobacterial CF_O_ assembly factor Atp1 and acquired an N-terminal domain with a distinct function in the green lineage.

### AtCGL160N recruits a stromal α_3_β_3_γε complex for late CF_1_-CF_O_ assembly steps

Despite structural similarities and comparable subunit compositions, the number of known assembly factors for ATP synthases is markedly higher in chloroplasts than in bacterial systems (reviewed in Zhang et al., 2020). Thus, the expanded molecular inventory for CF_1_-CF_O_ assembly in chloroplasts might reflect the need for tight post-translational control of CF_1_-CF_O_ formation, since the complex plays a central role in *pmf* utilization and regulation of photosynthesis (reviewed in Avenson et al., 2005). In this context, an important finding of previous studies was that disruption of full-length AtCGL160 (Rühle et al., 2014; Fristedt et al., 2015) was more detrimental to levels of functional ATP synthase than the loss of Atp1/UncI in non-photosynthetic bacteria (Gay, 1984; Liu et al., 2013). Furthermore, we show here that expression of *P_35S_:AtCGL160C* in plants that lack AtCGL160N only partially restores CF_1_-CF_O_ levels and activity (Figure 4). These observations prompted us to investigate the molecular function of the green-lineage-specific AtCGL160N in the CF_1_-CF_O_ assembly process in more detail.

Several lines of evidence suggest that the N-terminal domain of AtCGL160 recruits a stromal CF_1_ intermediate, while the C-terminal segment participates in c_14_-ring assembly: (1) AtCGL160N protrudes into the stroma, as deduced from protease protection assays (Figure 1); (2) formation of the c_14_ ring is restored in the presence of AtCGL160C alone, but CF_1_ accumulates strongly in the stroma in the absence of AtCGL160N (Figure 5); (3) CF_1_ subunits are differentially enriched in co-IP analyses performed with solubilized thylakoids isolated from *P_35S_:AtCGL160* plants (Figure 6, A and B); (4) AtCGL160 co-migrates with a large complex after DSP-mediated crosslinking (Figure 6C); and (5) AtCGL160N interacts with CF_1_-β in yeast two-hybrid experiments (Figure 7).

A role for AtCGL160 in the incorporation of CF_1_ into the holocomplex was previously proposed by Fristedt et al. (2015). This assumption was based on the observations that AtCGL160 co-migrated with CF_1_ subcomplexes in BN/SDS-PAGE analyses and could be cross-linked to CF_1_ subunits in WT protein samples. However, we detected AtCGL160 predominantly in the monomer fraction in untreated thylakoid preparations in this study (Figure 6C), as well as in previous work (Rühle et al., 2014), and co-migration of AtCGL160 with high-molecular-mass complexes was only observed after thylakoid proteins had been crosslinked with DSP (Figure 6C). Furthermore, the commercially available AtCGL160 antibody (AS12 1853, Agrisera) employed in the study of Fristedt et al. (2015) was found here to cross-react strongly with CF_1_-α or CF_1_-β (Supplemental Figure S3A), which complicates the interpretation of one-dimensional co-migration and crosslinking experiments in the absence of appropriate controls. Therefore, a new antibody was generated that does not cross-react with CF_1_-CF_O_ subunits and thus provides a reliable means of probing the molecular interactions of AtCGL160 (Supplemental Figure S3B).

In addition to CGL160, ALB4 was previously suggested to be involved in joining a CF_1_ to a CF_O_ module (Benz et al., 2009). Another study provided evidence that ALB4 and its paralog ALB3 physically interact with each other and show significant functional overlap in the membrane insertion of subunits of the Cyt *b_6_f* complex (Trosch et al., 2015). Moreover, alleles of *ALB4* (or alternatively *SUPPRESSOR OF TIC40 LOCI 1, STIC1*) have been identified as suppressors of the chloroplast protein import mutant *tic40* (Bedard et al., 2017), and ALB4/STIC1 and SUPPRESSOR OF TIC40 LOCI 2 (STIC2) were shown to act together in thylakoid protein targeting in a pathway that also involves chloroplast SIGNAL RECOGNITION PARTICLE 54 (cpSRP54) and a chloroplast homolog of the bacterial SRP receptor FtsY (cpFtsY). In our study, we did not identify ALB4/STIC1 in co-IP experiments with anti-AtCGL160 antibodies (Figure 6, Supplemental Table S1), and amounts of thylakoid-associated CF_1_-β in *atalb4-1* mutants (SALK_136199C) grown under short-day conditions were unaltered (Supplemental Figure S7). Thus, ALB4/STIC1 does not act in concert with CGL160 in late stages of CF_1_-CF_O_ assembly but serves as a general thylakoid protein biogenesis factor involved in folding or assembly of a specific subset of transmembrane proteins (Bedard et al., 2017).

### AtCGL160 is critical for chloroplast development in the dark

It has long been thought that the hydrolytic activity of CF_1_-CF_O_ needs to be inactivated in the dark to prevent futile ATP depletion (Ort and Oxborough, 1992). However, analysis of the constitutively redox-activated γ-subunit mutant *gamera*, in which a “dark *pmf*” is maintained, revealed increased stability of photosynthetic complexes upon prolonged darkness, suggesting that a certain degree of ATPase activity may be beneficial during the night (Kohzuma et al., 2017). Concomitantly, several processes have been proposed to depend on the maintenance of a dark *pmf*. These include thylakoid protein transport via the Tat- and Sec-dependent pathways, modulation of protease activity and ion homeostasis in the chloroplast. In this regard, a remarkable influence of AtCGL160 disruption on leaf variegation (Figure 2) and chloroplast development (Figure 3) was observed exclusively under short-day conditions. Surprisingly, this phenotype was not detectable in *atcgld11-1* plants with a defect in CF_1_ assembly and reduced amounts of CF_1_-CF_O_ compared to those in *atcgl160-1* (Figure 4E). However, the leaf phenotype correlated with the accumulation of a CF_1_ intermediate in the stroma (Figure 5C). Thus, AtCGL160-mediated CF_1_ recruitment might also be critical in preserving the dark *pmf* at night. Alternatively, stroma-enriched CF_1_ complexes (Figure 5C) could alter the chloroplast ATP/ADP ratio by excessive hydrolytic activity, and disturb ATP-dependent nocturnal processes that ultimately lead to premature chloroplast degradation (Figure 3).

### AtCGL160 is a central CF_1_-CF_O_ assembly factor with multiple functions

**Figure 9:**
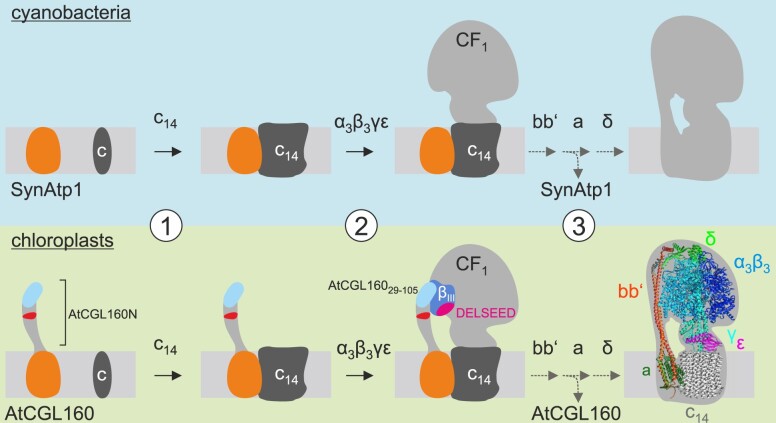
SynAtp1 and AtCGL160 in CF_1_-CF_O_ assembly. (1) SynAtp1 (upper panel) and AtCGL160 (lower panel) operate both in c_14_ assembly. (2) Recruitment of a stromal CF_1_ intermediate (α_3_β_3_γε) is assisted by the N-terminal domain AtCGL160N specific for the green lineage. Interaction is mediated by AtCGL160_29–105_ (light blue) and subdomain CF_1_-β_III_ (blue), while the phosphorylatable segment 106–134 aa (in red) is dispensable for interaction. CF_1_-β_III_ contains the conserved DELSEED motif (purple). (3) The exact timing of assembly factor release is unknown. However, SynAtp1 and AtCGL160 interacted with CF_O_-b in split-ubiquitin assays (Rühle et al., 2014) and might stay attached to a CF_1_-CF_O_ assembly intermediate until CF_O_-a or CF_1_-δ incorporation. The atomic model of spinach CF_1_-CF_O_ was retrieved from the PDB database (ID: 6fkh, Hahn et al. 2018) and then formatted using ChimeraX (Pettersen et al., 2021).

Assembly of membrane-embedded ATP synthase modules and their subsequent association with F_1_ subcomplexes are critical steps in bacterial and organellar ATP synthase biogenesis, as premature formation of the proton-translocating channel between the c-ring and the a-subunit (equivalent to the ATP9 ring and the ATP6 subunit in mitochondria) can lead to uncontrolled dissipation of the *pmf* (Birkenhäger et al., 1999; Franklin et al., 2004), and only efficient integration of F_1_ triggers ATP production. In this context, molecular aspects of the assembly processes were recently elucidated for bacterial (reviewed in Deckers-Hebestreit, 2013), as well as yeast and human mitochondrial ATP synthases (reviewed in Song et al., 2018). One significant outcome was that, while ATP synthase assembly pathways and the repertoire of auxiliary factors differ among these systems, formation of the proton-translocating unit during the final assembly steps is common to all of them. Intriguingly, our data revealed a dual involvement of AtCGL160 in CF_1_-CF_O_ assembly, namely in c-ring formation and the recruitment of a CF_1_ intermediate (Figure 9). In fact, these two events were suggested to proceed sequentially in the assembly of bacterial ATP synthases (Deckers-Hebestreit, 2013). Since an *E. coli* strain lacking subunit δ accumulates a c_10_α_3_β_3_γε subcomplex, it is assumed that cytoplasmic F_1_ first binds to the c_10_ ring, and c_10_α_3_β_3_γε associates with the ab_2_ module in a δ-dependent manner in the final assembly step (Hilbers et al., 2013).

By analogy with the bacterial assembly pathway, AtCGL160 may facilitate the integration of a stator assembly module into the holo-complex. Indeed, the interaction of AtCGL160C and SynAtp1 with CF_O_-b has been demonstrated in split-ubiquitin assays (Rühle et al., 2014). Moreover, CF_O_-a was less highly enriched in co-IP analyses than other CF_1_-CF_O_ subunits (Figure 6, A and B), which might argue for the release of AtCGL160 after functional incorporation of CF_O_-a in the final steps of CF_1_-CF_O_ assembly (Figure 9). In this scenario, AtCGL160 could act as a placeholder to prevent the premature formation of proton-translocating intermediates. A similar function has been described for the inner membrane assembly (INA) complex in yeast mitochondria, which binds to the c-ring, but also to a distinct assembly intermediate consisting of ATP6, ATP8, ATP10, ATP23, peripheral stalk subunits, and the F_1_ domain (Naumenko et al., 2017). This ensures that the c-ring and subunit ATP6 are assembled into the proton-conducting unit in a controlled manner. However, due to a generally low turnover rate of CF_1_-CF_O_ assembly (reviewed in Schöttler et al., 2014) and inefficient detection of distinct thylakoid-integral intermediates, a robust CF_O_ assembly map is still lacking, and “true” stator-containing assembly modules have not been described so far.

Nevertheless, a straightforward assembly mechanism for the recruitment of CF_1_ can be derived from our study (Figure 9). After AtCGL160-assisted ring formation (Rühle et al., 2014), the stromally oriented AtCGL160N (Figure 1) binds to a CF_1_ intermediate consisting of α_3_β_3_γε but not subunit δ (Figure 5C, Supplemental Figure S5). Recruitment is mediated through interaction of AtCGL160_29–105_ with subunit CF_1_-β; thus, the phosphorylatable AtCGL160 segment is dispensable for the interaction (Figure 7). Since AtCGL160 can be cross-linked to high-molecular-mass complexes that are larger than CF_1_ (Figure 6C), AtCGL160 might remain attached to a putative c_14_α_3_β_3_γε or bb′c_14_α_3_β_3_γε intermediate. Its release could then be triggered by the incorporation of subunit CF_O_-a or CF_1_-δ in the final assembly steps.

At this stage, we cannot rule out the possibility that AtCGL160N might have regulatory functions beyond CF_1_ recruitment, as it interacts with the thylakoid-proximal domain III of CF_1_-β, which contains the conserved DELSEED motif (Figure 7B). Several regulatory mechanisms have been elucidated in which the subunit β and the DELSEED motif are implicated. For instance, the autoinhibitory subunit ε interacts with the DELSEED motif in bacteria (Tanigawara et al., 2012; Sobti et al., 2016), whereas in bovine (Cabezon et al., 2003) and yeast mitochondria (Robinson et al., 2013), the small protein IF_1_ inhibits ATPase activity by binding at the α/β interface. In plants, a regulatory mechanism controls CF_1_-CF_O_ activity also involving the DELSEED and an L-shaped, two β-hairpin containing motif with two conserved redox-sensitive cysteines in the CF_1_-γ subunit (Hahn et al., 2018). By analogy with the role of IF_1_, which was shown to inhibit ATPase activity during the assembly of human mitochondrial ATP synthases (He et al., 2018), AtCGL160N may regulate ATPase activity during CF_1_-CF_O_ assembly via an as yet unknown mechanism.

## Methods

### Bioinformatics sources

Protein and gene sequences were downloaded from the Arabidopsis Information Resource server (TAIR; http://www.arabidopsis.org), Phytozome (https://phytozome.jgi.doe.gov/pz/portal.html), and the National Center for Biotechnology Information server (NCBI; http://www.ncbi.nlm.nih.gov/). TPs were predicted by ChloroP (http://www.cbs.dtu.dk/services/ChloroP/; Emanuelsson et al., 1999). Structural data were obtained from the PDB homepage (https://www.rcsb.org/) and processed with ChimeraX (https://www.cgl.ucsf.edu/chimerax/; Pettersen et al., 2021). Multiple sequence alignments were generated with the CLC workbench software (v8.1) and protein features were visualized with Protter (https://wlab.ethz.ch/protter/start/; Omasits et al., 2014). Co-migration of stromal proteins was examined with the online tool PCom-DB (http://pcomdb.lowtem.hokudai.ac.jp/proteins/top; Takabayashi et al., 2017). Boxplots were drawn with BoxPlotR (http://shiny.chemgrid.org/boxplotr/; Spitzer et al., 2014).

### Plant material and growth conditions

Arabidopsis (*A. Thaliana*) T-DNA lines for *atcgl160-1* (SALK_057229, Col-0 background*)*, *atcgld11-1* (SALK_019326C, Col-0 background), and *atalb4-1* (SALK_136199C) were obtained from the SALK collection (Alonso et al., 2003). Plants were grown in potting soil (A210, Stender, Schermbeck, Germany) under controlled greenhouse conditions (70–90 µmol photons m^−2^ s^−1^, 16/8-h light/dark cycles), or in climate chambers (equipped with 17-W cool white fluorescent lamps; CLF Plant Climatics) on an 8-h light/16-h dark cycle for biochemical and physiological analyses. Fertilizer was added to plants grown under greenhouse conditions according to the manufacturer’s recommendations (Osmocote Plus; 15% nitrogen [w/v], 11% [w/v] P_2_O_5_, 13% [w/v] K_2_O, and 2% [w/v] MgO; Scotts, Germany). Plants were watered with tap water. For domain-specific complementation assays, either the complete coding region of *AtCGL160* (*P_35S_:AtCGL160*) or parts of the CDS corresponding to amino acids 1–206 (*P_35S_:AtCGL160N*) and 207–350 (*P_35S_:AtCGL160C*) were cloned into the binary Gateway vector pB2GW7 (Karimi et al., 2002), placing the genes under control of the cauliflower mosaic virus (CaMV) 35S promoter. The putative, TP coding sequence (for amino acids 1–46) was fused to the *AtCGL160C* CDS in the case of the *P_35S_:AtCGL160C* vector. For the cross-species complementation construct, the codon usage of the *synatp1* CDS (UniProt ID P27196) was optimized for expression in Arabidopsis (IDT, Coralville, IA, USA) and fused to the *AtCGL160N* sequence by fusion PCR. The constructs were first transformed into *Agrobacterium tumefaciens* strain GV3101, and then into *atcgl160-1* plants by the floral-dip method (Clough and Bent, 1998). T1 plants were selected by screening for Basta resistance. Basta positives were screened for equal amounts of the *AtCGL160* transcript by RNA gel-blot hybridization or immunodetection of AtCGL160N as described below.

### Transmission electron microscopy

Leaf pieces of about 1.5 × 1.0 mm were cut with a new double edge razor blade (Feather, Osaka, Japan) and immediately immersed in fixation buffer (0.1 M sodium phosphate buffer, pH 7.4, 2.5% [v/v] glutaraldehyde, 4% [v/v] formaldehyde) at room temperature. A mild vacuum (about 20 mbar) was applied until the leaf pieces sank, the fixation buffer was replaced with fresh one and the samples were fixed overnight at 4°C. After three 10-min washes in sodium phosphate buffer (pH 7.4), the samples were osmicated with 1% osmium tetroxide and 1.5% potassium ferricyanide in 0.1 M sodium phosphate buffer (pH 7.4) for 60 min at 4°C. The samples were rinsed 3 times for 10 min each time in distilled water and incubated in 1% uranyl acetate (in distilled water) at 4°C overnight. After three washes of 10 min each in distilled water the samples were dehydrated using increasing concentrations of ethanol and infiltrated, with propylene oxide as an intermediate solvent, in glycid ether 100 (formerly Epon 812; Serva, Heidelberg, Germany) following standard procedures. Polymerization was carried out for 40–48 h at 65°C. Ultrathin sections (∼60 nm) were cut with a diamond knife (type ultra 35°; Diatome, Biel, Suisse) on an EM UC7 ultramicrotome (Leica Microsystems, Wetzlar, Germany) and mounted on single-slot Pioloform-coated copper grids (Plano, Wetzlar, Germany). The sections were stained using uranyl acetate and lead citrate (Reynolds, 1963) and viewed with a JEM-1400 Plus transmission electron microscope (JEOL, Tokyo, Japan) operated at 80 kV. Micrographs were taken using a 3.296 × 2.472 pixel charge-coupled device camera (Ruby, JEOL). The number of grana was determined in 25 chloroplasts per genotype from green leaf sections, where a granum was defined by at least two stacked thylakoid membranes. The grana height was analyzed using 417 (Col-0), 402 (*atcgl160-1*), and 406 (*P_35S_:AtCGL160C*) grana.

### Chl *a* fluorescence measurements

*In vivo* Chl *a* fluorescence of whole plants was measured using an imaging Chl fluorometer (Imaging PAM, Walz, Effeltrich, Germany). Plants were dark-adapted for 20 min and exposed to a pulsed, blue measuring beam (4 Hz, intensity 3, gain 3, damping 2; F_O_) and a saturating light flash (intensity 10) to calculate Fv/Fm. PSII quantum yield [Y(II) = (Fm′ − F)/Fm′] and NPQ [NPQ = (Fm − Fm′)/Fm′] for Col-0, *atcgl160-1*, *P_35S_:AtCGL160, P_35S_:AtCGL160N, P_35S_:AtCGL160C*, and *atcgld11-1* plants were determined in light saturation curve analyses by gradually (every 180 s) increasing the light intensity (0, 20, 55, 110, 185, 280, 335, 395, 460, 530, 610, 700 µmol photons m^−2^ s^−1^), with only the data at 185 µmol photons m^−2^ s^−1^ presented in this study (Figure 4A). Y(II) and NPQ of *P_35S_:AtCGL160N-SynATP1* and control plants were recorded 600 s after light induction experiments (110 µmol photons m^−2^ s^−1^; Figure 8, B–D).

### ECS measurements

ECS measurements were performed using the Dual-PAM-100 (Walz, Effeltrich, Germany) equipped with a P515/535 emitter-detector module (Schreiber and Klughammer, 2008). The measurement was carried out at 23°C under ambient CO_2_ conditions. Plants grown in short-day conditions for 4 weeks were light-adapted, and detached leaves were illuminated for at least 10 min with 129 µmol photons m^−2^ s^−1^ red light. After illumination, dark-interval relaxation kinetics (DIRK) were measured in the millisecond to second range. Values for *pmf* (ECSt), and proton conductivity ($g_{H}^{+}$) were calculated as described (Cruz et al., 2001; Schreiber and Klughammer, 2008). Briefly, the maximum amplitude of the inverse electrochromic band-shift kinetic was measured in the second range and normalized to a single saturating P515 pulse (ECS_ST_) measured after 4 min of dark incubation. For proton conductivity, electrochromic band-shift kinetics were recorded in the millisecond range in five consecutive periods of darkness (2 s), separated by light intervals of 30 s (Schreiber and Klughammer, 2008). Averaged signals were fitted to a single exponential decay function and the reciprocal value of the ECS decay time constant was used to estimate the proton conductivity $g_{H}^{+}$ (Kanazawa and Kramer, 2002). Proton flux (ν_H_^+^) was determined from the initial rate of decay (1–20 ms) of the ECS signal.

### AtCGL160 antibody generation

Rabbit antibodies were generated against AtCGL160 that had been heterologously expressed in *Escherichia coli* and then purified. To this end, the coding sequence corresponding to AtCGL160_29–206_ was cloned into the pMal-c5x vector (New England Biolabs) and purified on amylose columns (New England Biolabs) according to the manufacturer’s instructions. The protein was injected into rabbits for antibody production (Pineda, Berlin, Germany). To reduce epitope cross-reactions, the antiserum was purified on a column crosslinked with heterologously expressed AtCGL160_29–206_ fused to the glutathione-S-transferase (GST) tag. Purified antibody was employed at a dilution of 1:1,000. Signals were detected by enhanced chemiluminescence (Pierce™ ECL Western Blotting Substrate, Thermo Scientific) using an ECL reader system (Fusion FX7; PeqLab, Erlangen, Germany).

### Nucleic acid analysis

Total RNA from snap-frozen leaves was extracted with an RNeasy Plant Mini Kit (Qiagen) according to the supplier’s instructions. Samples equivalent to 20 µg total RNA were fractionated by electrophoresis in formaldehyde-containing agarose gels (1.2%), blotted onto nylon membranes (Hybond-N+, Amersham Bioscience) and fixed by UV radiation (Stratalinker UV Crosslinker 1800). To control for equal loading, abundant RNAs on nylon membranes were stained with methylene blue solution (0.02% [w/v] methylene blue, 0.3 M sodium acetate pH 5.5). To detect gene-specific transcripts, DNA fragments amplified from cDNA were labelled with radioactive [α-^32^P]dCTP and subsequently used as probes in hybridization experiments (see Supplemental Table S2 for primer information). Signals were detected with the Typhoon Phosphor Imager System (GE Healthcare).

### Protein analysis

Leaves from 4-week-old plants grown under short-day conditions were harvested shortly after the onset of the light period, and thylakoid membrane-enriched samples were isolated according to Rühle et al. (2014). Crosslinking of thylakoids was performed by incubation with 2.5 mM DSP (Thermo Scientific). After incubation for 20 min on ice, crosslinking was quenched with 60 mM Tris/HCl (pH 7.5). Chl concentrations were determined as described in Porra et al. (1989). For immunotitrations, thylakoid membrane pellets were resuspended in loading buffer (100 mM Tris/HCl pH 6.8, 50 mM dithiothreitol [DTT], 8% [w/v] SDS, 24% [w/v] glycerol and 0.02% [w/v] bromophenol blue). Denaturation for 5 min at 70°C and protein fractionation on Tricine-SDS-PAGE gels (10% gels supplemented with 4M urea) was carried out according to Schägger (2006). Immunodetections were performed as described below. Sample preparation for BN-PAGE was performed with freshly prepared thylakoids as described in Peng et al. (2008). First, membranes were washed twice in wash buffer (20% glycerol, 25 mM BisTris/HCl pH 7.0). Then, samples were treated with wash buffer containing 1% (w/v) n-dodecyl β-D-maltoside and adjusted to 1 mL mg^−1^ Chl for 10 min on ice. After centrifugation (16,000 × *g*, 20 min, 4°C), supernatants were supplemented with 1/10 volume of BN sample buffer (100 mM BisTris/HCl pH 7.0, 750 mM ε-aminocaproic acid, 5% [w/v] Coomassie G-250). BN-PAGE gels (4%–12% gradient) were prepared as described in Schägger et al., (1994). Solubilized samples corresponding to 60 µg Chl were loaded per lane and gels were run at 4°C overnight. To separate complexes into their subunits, BN-PAGE strips were treated with denaturing buffer (0.2 M Na_2_CO_3_, 5% [w/v] SDS, 50 mM DTT) for 30 min at room temperature and loaded on Tricine-SDS-PAGE gels. Gels were subsequently subjected to immunoblot analysis with antibodies against CF_1_-CF_O_ subunits and AtCGL160, as described below.

For analysis of the stromal CF_1_ intermediate, intact chloroplasts from 4-week-old plants were isolated according to Rühle et al. (2021). After lysis in 25 mM HEPES/KOH (pH 7.5) containing 5 mM MgCl_2_ for 30 min on ice, the stromal fraction was separated from membranes by centrifugation at 35,000 × *g* for 30 min (4°C). Protein concentration was measured using the Bradford Protein Assay (Bio-Rad). Stromal BN analysis was performed according to Reiter et al. (2020). In brief, chloroplast-enriched pellets were resuspended in BN washing buffer and mechanically disrupted by passage through an 0.45-mm syringe. The stromal fraction was separated from membranes by centrifugation at 35,000 × *g* for 30 min (at 4°C). Total soluble protein (100 µg) was mixed with 1/10 volume of BN sample buffer before fractionation in the first dimension as described above.

### Immunoblot analyses

Proteins fractionated by gel electrophoresis were transferred to polyvinylidene difluoride membranes (PVDF; Immobilon-P, Millipore) using a semi-dry blotting system (BioRad) as described in the supplier’s instructions. After blocking with TBST (10 mM Tris/HCl pH 8.0, 150 mM NaCl and 0.1% [v/v] Tween-20) supplemented with 3% (w/v) skim milk powder, the membranes were incubated with antibodies at 4°C overnight. Antibodies used in this study were obtained from Agrisera (CF_1_-β: AS05 085, 1:5,000; CF_1_-γ: AS08 312, 1:5,000; CF_O_-b: AS10 1604, 1:5,000; CF_O_-c: AS09 591, 1:3,000; AtCGL160: AS12 1853, 1:1,000; PsbO: AS05 092, 1:10,000 and PsaD: AS09 461, 1:1,000).

### Yeast-two-hybrid experiments

Yeast-two-hybrid assays were carried out using the Matchmaker Two-Hybrid System Kit (Clontech). The *AtCGL160_29–206aa_* CDS without the signal peptide (see Supplemental Table S2 for primer information) was cloned into the bait vector pGBKT7 (BD-AtCGL160N), whereas the coding sequences of CF_1_-α, -β, -γ, -δ, -ε, the soluble domains of CF_O_-b (51–184 aa) and b' (109–219 aa), AtCGL160N and the CF_1_ assembly factor AtCGLD11 were cloned into the prey vector pGADT7 (named AD-CF_1_-α, AD-CF_1_-β, AD-CF_1_-γ, AD-CF_1_-δ, AD-CF_1_-ε, AD-CF_O_-b, AD-CF_O_-b', AD-AtCGL160N, and AD-AtCGLD11). As in the case of AtCGL160, signal peptide sequences were omitted from the nucleus-encoded subunits CF_1_-γ, CF_1_-δ, CF_O_-b', and AtCGLD11. For testing interaction between AtCGL160N and CF_1_-β in a reciprocal approach, the CDS of CF_1_-β was also cloned into the bait vector pGBKT7 (BD-CF_1_-β). For binding-domain analysis of CF_1_-β, the respective CDS was subdivided into three parts, according to Groth and Pohl (2001), and cloned into pGADT7. In the case of AtCGL160N binding-site analysis, sequences coding for 29–73, 74–105, 106–134, 135–160, and 161–206 aa were deleted from the BD-AtCGL160 vector using a site-directed mutagenesis kit (NEB). Primers are listed in Supplemental Table S2. Bait and prey vectors were co-transformed into AH109 yeast strains (Clontech) following the manufacturer’s instructions. Co-transformants were selected on synthetic dropout (SD) medium (Clontech) lacking leucine and tryptophan (-LT). In order to identify protein interactions, double transformants were grown on SD medium lacking leucine, tryptophan, histidine, and adenine (-LTHA).

### Co-immunoprecipitation

Freshly extracted thylakoids corresponding to ∼10 mg chlorophyll were resuspended in 500 µL extraction buffer (50 mM Tris/HCl pH 7.5, 150 mM NaCl, 1 mM MgCl_2_, 5% [w/v] glycerol, 1% [v/v] Nonidet P40 [NP40], 0.2 mM phenylmethylsulfonyl fluoride [PMSF]) and solubilized for 30 min on ice. After centrifugation at 35,000 × *g* for 30 min and 4°C, the supernatant was added to 20 µL Dynabeads (Thermo Scientific), equilibrated with equilibration buffer (50 mM Tris/HCl pH 7.5, 150 mM NaCl, 5% [w/v] glycerol, 0.05% [v/v] NP40) and labelled with AtCGL160 antibody according to the manufacturer’s instructions. The suspension was incubated with rotation for 3 h at 4°C, washed 3 times with equilibration buffer, and twice with the same buffer but omitting NP40. Proteins were eluted with 100 µL 0.1 M glycine pH 2.0 for 10 min and neutralized with 100 µL 0.1 M ammonium bicarbonate. After treatment with 10 µL of 45 mM DTT and 10 µL of 0.1 M iodoacetamide, samples were digested with 1.5 µg of trypsin at 37°C overnight. Peptides were desalted with home-made C18 stage tips (Rappsilber et al., 2003), vacuum-dried to near dryness and stored at –80°C. LC MS/MS run and data analysis were performed as described in Reiter et al. (2020). The mass spectrometry proteomics data have been deposited to the ProteomeXchange Consortium via the PRIDE (Perez-Riverol et al., 2022) partner repository with the dataset identifier PXD032230.

### Generation and analysis of *Synechocystis* sp. PCC 6803 strains

Glucose-tolerant *Synechocystis* sp. PCC 6803 (Synechocystis) WT and mutant strains were grown in BG11 medium (Rippka et al., 1979) optionally supplemented with 5 mM glucose. Liquid cultures were kept at 30°C under continuous illumination (∼60 µmol photons m^−2^ s^−1^) on shakers (120 rpm). Cultures of *P_psbA2_:synatp1* and *P_psbA2_:Δsynatp1* strains were supplemented with 50 µg mL^−1^ kanamycin. Synechocystis strain *P_psbA2_:synatp1* and *P_psbA2_:Δsynatp1* were generated by homologous recombination. Constructs were cloned using the Golden Gate cloning strategy (Engler et al., 2008) and assembled into the destination vector pICH69822 (Icon Genetics GmbH, Halle, Germany). Selection of positive transformants and segregation were conducted in the presence of increasing kanamycin concentrations (up to 500 µg mL^−1^). DNA extraction and segregation analyses were carried out using the PHIre Plant Direct PCR-Kit according to the manufacturer’s instructions (Thermo Fisher Scientific, Waltham, Massachusetts, USA). Total RNA was isolated from mixotrophically grown cells as previously described (Dann and Leister, 2019). RNA concentration was determined using a Nanodrop 2000 spectrophotometer (PeqLab, Erlangen, Germany). After adjusting RNA concentration and examination of RNA integrity by gel electrophoresis, samples were treated with DNase according to the manufacturer’s instructions (RNase-free DNase I, New England Biolabs). Synthesis of cDNA was carried out with 250 ng RNA using the iScript cDNA Synthesis Kit (BioRad, Munich, Germany). Quantitative real-time PCR analysis with primers described in Supplemental Table S2 was performed on a BioRad CFX Connect Real-Time system with the iQ SYBR Green Supermix (BioRad). Expression of *synatp1* was quantified in technical triplicates, normalized to *rrn16S* expression (Pinto et al., 2012) and referred to *synatp1* expression of the wildtype. Oxygen consumption and production rates were determined with the Clark-type oxygen electrode Oxytherm+ system (Hansatech Instruments Ltd, UK) at 30°C. In brief, oxygen uptake rates (nmol O_2_ min^−1^${\text{OD}}_{730}^{-1}$) of cell cultures at the end of their exponential growth phase (OD_730_∼0.2–0.4) were recorded for 10 min in the dark. Photosynthetic activity was determined by measuring the oxygen production rate (nmol O_2_ min^−1^${\text{OD}}_{730}^{-1}$) at 400 µmol photons m^−2^ s^−1^ for 5 min. Amounts of thylakoid ATP synthase in *P_psbA2_:synatp1* and *P_psbA2_:Δsynatp1* were analyzed by immunodetection. Thylakoids were isolated as described (Gandini et al., 2017), adjusted to 2 µg Chl *a* (which corresponds to 100% in Supplemental Figure S8), and subjected to Tricine-SDS-PAGE and immunodetection assays as outlined in the main text.

## Accession numbers

AtCGL160 (AT2G31040, UniProt identifier O82279), AtCGLD11 (At2G21385), AtALB4/AtSTIC1 (AT1G24490), SynAtp1 (Sll1321)

## Supplemental data

The following materials are available in the online version of this article.

**Supplemental Figure S1.** Multiple alignment of the N-terminal portions of CGL160 sequences identified in species belonging to the green lineage.

**Supplemental Figure S2.** Screening of *P_35S_:AtCGL160, P_35S_:AtCGL160C*, and *P_35S_:AtCGL160N* plants.

**Supplemental Figure S3.** Immunodetection of AtCGL160 in Col-0, *atcgl160-1*, *P_35S_:AtCGL160, P_35S_:AtCGL160N, P_35S_:AtCGL160C*, and *atcgld11-1* plants.

**Supplemental Figure S4.** Quantification of grana number and height in Col-0, *atcgl160-1*, and *P_35S_:AtCGL160C* green leaf sector samples.

**Supplemental Figure S5.** Characterization of the stromal CF_1_ complex in *atcgl160-1* plants.

**Supplemental Figure S6.** Immunoblot analysis of AtCGL160 co-immunoprecipitation assays.

**Supplemental Figure S7.** Quantification of thylakoid-bound CF_1_-β subunits in *atalb4-1* Arabidopsis mutant lines.

**Supplemental Figure S8.** Interaction of BD-CF_1_-β and AD-AtCGL160N in yeast two-hybrid assays.

**Supplemental Figure S9.** Lack of segregation in a *synatp1* knockout strain of *Synechocystis* sp. PCC 6803 (Synechocystis) indicated an essential function for SynAtp1.

**Supplemental Figure S10.** The endogenous promoter of the *atp1* operon in Synechocystis could be functionally replaced by the strong *psbA2* promoter (*P_psbA2_*).

**Supplemental Table S1.** AtCGL160 co-immunoprecipitation experiments.

**Supplemental Table S2.** Primers used in this study.

**Supplemental Data Set S1.** Statistical analyses.

## Supplementary Material

koac306_Supplementary_Data

## Notes

B.R. and T.R. designed research. B.R., L.R., G.M., S.G., and T.R. carried out experiments. B.R., D.L., and T.R. prepared the article. T.R. supervised the whole study.

The author responsible for distribution of materials integral to the findings presented in this article in accordance with the policy described in the Instructions for Authors (https://academic.oup.com/plcell) is: Thilo Rühle (thilo.ruehle@biologie.uni-muenchen.de).

## References

[koac306-B1] Aigner H , Wilson RH, Bracher A, Calisse L, Bhat JY, Hartl FU, Hayer-Hartl M (2017) Plant RuBisCo assembly in *E. coli* with five chloroplast chaperones including BSD2. Science 358: 1272–1278 29217567 10.1126/science.aap9221

[koac306-B2] Alonso JM , Stepanova AN, Leisse TJ, Kim CJ, Chen H, Shinn P, Stevenson DK, Zimmerman J, Barajas P, Cheuk R, et al (2003) Genome-wide insertional mutagenesis of Arabidopsis thaliana. Science 301: 653–657 12893945 10.1126/science.1086391

[koac306-B3] Avenson TJ , Kanazawa A, Cruz JA, Takizawa K, Ettinger WE, Kramer DM (2005) Integrating the proton circuit into photosynthesis: progress and challenges. Plant Cell Environ 28: 97–109

[koac306-B4] Bedard J , Trosch R, Wu FJ, Ling QH, Flores-Perez U, Topel M, Nawaz F, Jarvisa P (2017) Suppressors of the chloroplast protein import mutant tic40 reveal a genetic link between protein import and thylakoid biogenesis. Plant Cell 29: 1726 28684427 10.1105/tpc.16.00962 PMC5559741

[koac306-B5] Benz M , Bals T, Gügel IL, Piotrowski M, Kuhn A, Schünemann D, Soll J, Ankele E (2009) Alb4 of Arabidopsis promotes assembly and stabilization of a non chlorophyll-binding photosynthetic complex, the CF1CF0-ATP synthase. Mol Plant 2: 1410–1424 19995738 10.1093/mp/ssp095

[koac306-B6] Birkenhäger R , Greie JC, Altendorf K, Deckers-Hebestreit G (1999) F0 complex of the Escherichia coli ATP synthase. Not all monomers of the subunit c oligomer are involved in F1 interaction. Eur J Biochem 264: 385–396 10491083 10.1046/j.1432-1327.1999.00652.x

[koac306-B7] Cabezon E , Montgomery MG, Leslie AG, Walker JE (2003) The structure of bovine F1-ATPase in complex with its regulatory protein IF1. Nat Struct Biol 10: 744–750 12923572 10.1038/nsb966

[koac306-B8] Chen GG , Jagendorf aT (1994) Chloroplast molecular chaperone-assisted refolding and reconstitution of an active multisubunit coupling factor CF1 core. Proc Natl Acad Sci USA 91: 11497–11501 7972091 10.1073/pnas.91.24.11497 PMC45258

[koac306-B9] Clough SJ , Bent AF (1998) Floral dip: a simplified method for Agrobacterium-mediated transformation of Arabidopsis thaliana. Plant J 16: 735–743 10069079 10.1046/j.1365-313x.1998.00343.x

[koac306-B10] Cruz JA , Sacksteder CA, Kanazawa A, Kramer DM (2001) Contribution of electric field (Delta psi) to steady-state transthylakoid proton motive force (pmf) in vitro and in vivo. control of pmf parsing into Delta psi and Delta pH by ionic strength. Biochemistry 40: 1226–1237 11170448 10.1021/bi0018741

[koac306-B11] Dann M , Leister D (2019) Evidence that cyanobacterial Sll1217 functions analogously to PGRL1 in enhancing PGR5-dependent cyclic electron flow. Nat Commun 10: 5299–5299 31757966 10.1038/s41467-019-13223-0 PMC6876563

[koac306-B12] Daum B , Nicastro D, Austin J, McIntosh JR, Kühlbrandt W (2010) Arrangement of photosystem II and ATP synthase in chloroplast membranes of spinach and pea. Plant Cell 22: 1299–1312 20388855 10.1105/tpc.109.071431 PMC2879734

[koac306-B13] Deckers-Hebestreit G (2013) Assembly of the Escherichia coli FoF1 ATP synthase involves distinct subcomplex formation. Biochem Soc Trans 41: 1288–1293 24059521 10.1042/BST20130096

[koac306-B14] Eberhard S , Loiselay C, Drapier D, Bujaldon S, Girard-Bascou J, Kuras R, Choquet Y, Wollman F-A (2011) Dual functions of the nucleus-encoded factor TDA1 in trapping and translation activation of atpA transcripts in Chlamydomonas reinhardtii chloroplasts. Plant J 67: 1055–1066 21623973 10.1111/j.1365-313X.2011.04657.x

[koac306-B15] Emanuelsson O , Nielsen H, von Heijne G (1999) ChloroP, a neural network-based method for predicting chloroplast transit peptides and their cleavage sites. Protein Sci 8: 978–984 10338008 10.1110/ps.8.5.978 PMC2144330

[koac306-B16] Engler C , Kandzia R, Marillonnet S (2008) A one pot, one step, precision cloning method with high throughput capability. PLoS One 3: e3647 18985154 10.1371/journal.pone.0003647 PMC2574415

[koac306-B17] Franklin MJ , Brusilow WSa, Woodbury DJ (2004) Determination of proton flux and conductance at pH 6.8 through single FO sectors from Escherichia coli. Biophys J 87: 3594–3599 15339819 10.1529/biophysj.104.044248 PMC1304824

[koac306-B18] Fristedt R , Martins NF, Strenkert D, Clarke CA, Suchoszek M, Thiele W, Schöttler MA, Merchant SS (2015) The thylakoid membrane protein CGL160 supports CF1CF0 ATP synthase accumulation in Arabidopsis thaliana. PLoS One 10: e0121658 25835989 10.1371/journal.pone.0121658 PMC4383579

[koac306-B19] Gandini C , Schmidt SB, Husted S, Schneider A, Leister D (2017) The transporter SynPAM71 is located in the plasma membrane and thylakoids, and mediates manganese tolerance in Synechocystis PCC6803. New Phytol 215: 256–268 28318016 10.1111/nph.14526

[koac306-B20] Gay NJ (1984) Construction and characterization of an Escherichia coli strain with a uncI mutation. J Bacteriol 158: 820–825 6327640 10.1128/jb.158.3.820-825.1984 PMC215515

[koac306-B21] Grahl S , Reiter B, Gügel Irene L, Vamvaka E, Gandini C, Jahns P, Soll J, Leister D, Rühle T (2016) The Arabidopsis protein CGLD11 is required for chloroplast ATP synthase accumulation. Mol Plant 9: 885–899 26979383 10.1016/j.molp.2016.03.002

[koac306-B22] Groth G , Pohl E (2001) The structure of the chloroplast F1-ATPase at 3.2 A resolution. J Biol Chem 276: 1345–1352 11032839 10.1074/jbc.M008015200

[koac306-B23] Hahn A , Vonck J, Mills DJ, Meier T, Kühlbrandt W (2018) Structure, mechanism, and regulation of the chloroplast ATP synthase. Science 360: eaat4318 29748256 10.1126/science.aat4318 PMC7116070

[koac306-B24] He J, Ford HC, , Carroll J, Douglas C, Gonzales E, Ding S, Fearnley IM, , Walker JE (2018) Assembly of the membrane domain of ATP synthase in human mitochondria. Proc Natl Acad Sci USA 115: 2988–2993 29440398 10.1073/pnas.1722086115 PMC5866602

[koac306-B25] Hilbers F , Eggers R, Pradela K, Friedrich K, Herkenhoff-Hesselmann B, Becker E, Deckers-Hebestreit G (2013) Subunit δ is the key player for assembly of the H(+)-translocating unit of Escherichia coli F(O)F1 ATP synthase. J Biol Chem 288: 25880–25894 23864656 10.1074/jbc.M113.484675 PMC3764793

[koac306-B26] Junge W , Nelson N (2015) ATP synthase. Annu Rev Biochem 84: 631–657 25839341 10.1146/annurev-biochem-060614-034124

[koac306-B27] Kanazawa A , Kramer DM (2002) In vivo modulation of nonphotochemical exciton quenching (NPQ) by regulation of the chloroplast ATP synthase. Proc Natl Acad Sci USA 99: 12789–12794 12192092 10.1073/pnas.182427499 PMC130538

[koac306-B28] Kanazawa A , Ostendorf E, Kohzuma K, Hoh D, Strand DD, Sato-Cruz M, Savage L, Cruz JA, Fisher N, Froehlich JE, et al (2017) Chloroplast ATP synthase modulation of the thylakoid proton motive force: implications for Photosystem I and Photosystem II photoprotection. Front Plant Sci 8: 1–12 28515738 10.3389/fpls.2017.00719 PMC5413553

[koac306-B29] Karimi M , Inze D, Depicker A (2002) GATEWAY vectors for Agrobacterium-mediated plant transformation. Trends Plant Sci 7: 193–195 11992820 10.1016/s1360-1385(02)02251-3

[koac306-B30] Karpowicz SJ , Prochnik SE, Grossman AR, Merchant SS (2011) The GreenCut2 resource, a phylogenomically derived inventory of proteins specific to the plant lineage. J Biol Chem 286: 21427–21439 21515685 10.1074/jbc.M111.233734 PMC3122202

[koac306-B31] Kohzuma K , Froehlich JE, Davis GA, Temple JA, Minhas D, Dhingra A, Cruz JA, Kramer DM (2017) The role of light–dark regulation of the chloroplast ATP synthase. Front Plant Sci 8: 1–14 28791032 10.3389/fpls.2017.01248 PMC5522872

[koac306-B32] Lill H , Nelson N (1991) The atp1 and atp2 operons of the cyanobacterium Synechocystis sp. PCC 6803. Plant Mol Biol 17: 641–652 1832989 10.1007/BF00037050

[koac306-B33] Liu J , Hicks DB, Krulwich TA (2013) Roles of AtpI and two YidC-type proteins from alkaliphilic Bacillus pseudofirmus OF4 in ATP synthase assembly and nonfermentative growth. J Bacteriol 195: 220–230 23123906 10.1128/JB.01493-12 PMC3553844

[koac306-B34] Mao J , Chi W, Ouyang M, He B, Chen F, Zhang L (2015) PAB is an assembly chaperone that functions downstream of chaperonin 60 in the assembly of chloroplast ATP synthase coupling factor 1. Proc Natl Acad Sci USA 112: 4152–4157 25775508 10.1073/pnas.1413392111 PMC4386354

[koac306-B35] Merchant SS , Prochnik SE, Vallon O, Harris EH, Karpowicz SJ, Witman GB, Terry A, Salamov A, Fritz-Laylin LK, Maréchal-Drouard L, et al (2007) The Chlamydomonas genome reveals the evolution of key animal and plant functions. Science 318: 245–250 17932292 10.1126/science.1143609 PMC2875087

[koac306-B36] Murphy BJ , Klusch N, Langer J, Mills DJ, Yildiz O, Kühlbrandt W (2019) Rotary substates of mitochondrial ATP synthase reveal the basis of flexible F1-Fo coupling. Science 364: eaaw9128 10.1126/science.aaw9128 31221832

[koac306-B37] Naumenko N , Morgenstern M, Rucktaschel R, Warscheid B, Rehling P (2017) INA complex liaises the F1Fo-ATP synthase membrane motor modules. Nat Commun 8: 1237 29093463 10.1038/s41467-017-01437-z PMC5665977

[koac306-B38] Obayashi T , Hayashi S, Saeki M, Ohta H, Kinoshita K (2009) ATTED-II provides coexpressed gene networks for Arabidopsis. Nucleic Acids Res 37: D987–D991 18953027 10.1093/nar/gkn807 PMC2686564

[koac306-B39] Omasits U , Ahrens CH, Müller S, Wollscheid B (2014) Protter: interactive protein feature visualization and integration with experimental proteomic data. Bioinformatics 30: 884–886 24162465 10.1093/bioinformatics/btt607

[koac306-B40] Ort DR , Oxborough K (1992) In situ regulation of chloroplast coupling factor activity. Annu Rev Plant Physiol Plant Mol Biol 43: 269–291

[koac306-B41] Ozaki Y , Suzuki T, Kuruma Y, Ueda T, Yoshida M (2008) UncI protein can mediate ring-assembly of c-subunits of FoF1-ATP synthase in vitro. Biochem Biophys Res Commun 367: 663–666 18182163 10.1016/j.bbrc.2007.12.170

[koac306-B42] Peng L , Shimizu H, Shikanai T (2008) The chloroplast NAD(P)H dehydrogenase complex interacts with photosystem I in Arabidopsis. J Biol Chem 283: 34873–34879 18854313 10.1074/jbc.M803207200 PMC3259898

[koac306-B43] Perez-Riverol Y , Bai J, Bandla C, Garcia-Seisdedos D, Hewapathirana S, Kamatchinathan S, Kundu DJ, Prakash A, Frericks-Zipper A, Eisenacher M, et al (2022) The PRIDE database resources in 2022: a hub for mass spectrometry-based proteomics evidences. Nucleic Acids Res 50: D543–D552 34723319 10.1093/nar/gkab1038 PMC8728295

[koac306-B44] Pettersen EF , Goddard TD, Huang CC, Meng EC, Couch GS, Croll TI, Morris JH, Ferrin TE (2021) UCSF ChimeraX: structure visualization for researchers, educators, and developers. Protein Sci 30: 70–82 32881101 10.1002/pro.3943 PMC7737788

[koac306-B45] Pfalz J , Bayraktar OA, Prikryl J, Barkan A (2009) Site-specific binding of a PPR protein defines and stabilizes 5′ and 3′ mRNA termini in chloroplasts. EMBO J 28: 2042–2052 19424177 10.1038/emboj.2009.121 PMC2718276

[koac306-B46] Pinto F, Pacheco CC, , Ferreira D, Moradas-Ferreira P, Tamagnini P (2012) Selection of suitable reference genes for RT-qPCR analyses in cyanobacteria. PloS one 7: e34983 22496882 10.1371/journal.pone.0034983 PMC3319621

[koac306-B47] Porra RJ , Thompson WA, Kriedemann PE (1989) Determination of accurate extinction coefficients and simultaneous equations for assaying chlorophylls a and b extracted with four different solvents: verification of the concentration of chlorophyll standards by atomic absorption spectroscopy. Biochim Biophys Acta 975: 384–394

[koac306-B48] Rappsilber J , Ishihama Y, Mann M (2003) Stop and go extraction tips for matrix-assisted laser desorption/ionization, nanoelectrospray, and LC/MS sample pretreatment in proteomics. Anal Chem 75: 663–670 12585499 10.1021/ac026117i

[koac306-B49] Reiland S , Messerli G, Baerenfaller K, Gerrits B, Endler A, Grossmann J, Gruissem W, Baginsky S (2009) Large-scale Arabidopsis phosphoproteome profiling reveals novel chloroplast kinase substrates and phosphorylation networks. Plant Physiol 150: 889–903 19376835 10.1104/pp.109.138677 PMC2689975

[koac306-B50] Reiland S , Finazzi G, Endler A, Willig A, Baerenfaller K, Grossmann J, Gerrits B, Rutishauser D, Gruissem W, Rochaix J-D, et al (2011) Comparative phosphoproteome profiling reveals a function of the STN8 kinase in fine-tuning of cyclic electron flow (CEF). Proc Natl Acad Sci USA 108: 12955–12960 21768351 10.1073/pnas.1104734108 PMC3150903

[koac306-B51] Reiter B , Vamvaka E, Marino G, Kleine T, Jahns P, Bolle C, Leister D, Rühle T (2020) The Arabidopsis protein CGL20 is required for plastid 50S ribosome biogenesis. Plant Physiol 182: 1222–1238 31937683 10.1104/pp.19.01502 PMC7054867

[koac306-B52] Reynolds ES (1963) Use of lead citrate at high Ph as an electron-opaque stain in electron microscopy. J Cell Biol 17: 208 13986422 10.1083/jcb.17.1.208 PMC2106263

[koac306-B53] Rippka R , Deruelles J, Waterbury JB, Herdman M, Stanier RY (1979) Generic assignments, strain histories and properties of pure cultures of cyanobacteria. J Gen Microbiol 111: 1–61

[koac306-B54] Robinson GC , Bason JV, Montgomery MG, Fearnley IM, Mueller DM, Leslie AG, Walker JE (2013) The structure of F(1)-ATPase from Saccharomyces cerevisiae inhibited by its regulatory protein IF(1). Open Biol 3: 120164 23407639 10.1098/rsob.120164 PMC3603450

[koac306-B55] Roitinger E , Hofer M, Kocher T, Pichler P, Novatchkova M, Yang J, Schlogelhofer P, Mechtler K (2015) Quantitative phosphoproteomics of the ataxia telangiectasia-mutated (ATM) and ataxia telangiectasia-mutated and rad3-related (ATR) dependent DNA damage response in Arabidopsis thaliana. Mol Cell Proteomics 14: 556–571 25561503 10.1074/mcp.M114.040352 PMC4349977

[koac306-B56] Rühle T , Leister D (2015) Assembly of F1F0-ATP synthases. Biochim Biophys Acta Bioenerg 1847: 849–860 10.1016/j.bbabio.2015.02.005 25667968

[koac306-B57] Rühle T , Dann M, Reiter B, Schünemann D, Naranjo B, Penzler J-F, Kleine T, Leister D (2021) PGRL2 triggers degradation of PGR5 in the absence of PGRL1. Nat Commun 12: 3941–3941 34168134 10.1038/s41467-021-24107-7 PMC8225790

[koac306-B58] Rühle T , Razeghi JA, Vamvaka E, Viola S, Gandini C, Kleine T, Schünemann D, Barbato R, Jahns P, Leister D (2014) The Arabidopsis protein CONSERVED ONLY IN THE GREEN LINEAGE160 promotes the assembly of the membranous part of the chloroplast ATP synthase. Plant physiol 1: 207–226 10.1104/pp.114.237883 PMC4012581 24664203

[koac306-B7386935] Schägger H, , Cramer WA, , von Jagow G (1994) Analysis of molecular masses and oligomeric states of protein complexes by blue native electrophoresis and isolation of membrane protein complexes by two-dimensional native electrophoresis. Analyt Biochem 217: 220–230 8203750 10.1006/abio.1994.1112

[koac306-B59] Schägger H (2006) Tricine-SDS-PAGE. Nat Protocols 1: 16–22 17406207 10.1038/nprot.2006.4

[koac306-B60] Schöttler MA, Tóth SZ, , Boulouis A, Kahlau S (2015) Photosynthetic complex stoichiometry dynamics in higher plants: biogenesis, function, and turnover of ATP synthase and the cytochrome b6f complex. J Exp Bot 66: 2373–2400 25540437 10.1093/jxb/eru495

[koac306-B61] Schreiber U , Klughammer C (2008) New accessory for the Dual-PAM-100: the P515/535 module and examples of its application. PAM Appl Notes 10: 1–10

[koac306-B62] Sobti M , Smits C, Wong AS, Ishmukhametov R, Stock D, Sandin S, Stewart AG (2016) Cryo-EM structures of the autoinhibited E. coli ATP synthase in three rotational states. Elife 5: 1–18 10.7554/eLife.21598 PMC5214741 28001127

[koac306-B63] Song J , Pfanner N, Becker T (2018) Assembling the mitochondrial ATP synthase. Proc Natl Acad Sci USA 115: 2850–2852 29514954 10.1073/pnas.1801697115 PMC5866614

[koac306-B64] Spitzer M , Wildenhain J, Rappsilber J, Tyers M (2014) BoxPlotR: a web tool for generation of box plots. Nat Methods 11: 121–122 24481215 10.1038/nmeth.2811 PMC3930876

[koac306-B65] Suzuki T , Ozaki Y, Sone N, Feniouk Ba, Yoshida M (2007) The product of uncI gene in F1Fo-ATP synthase operon plays a chaperone-like role to assist c-ring assembly. Proc Natl Acad Sci USA 104: 20776–20781 18083842 10.1073/pnas.0708075105 PMC2410078

[koac306-B66] Takabayashi A , Takabayashi S, Takahashi K, Watanabe M, Uchida H, Murakami A, Fujita T, Ikeuchi M, Tanaka A (2017) PCoM-DB update: a protein co-migration database for photosynthetic organisms. Plant Cell Physiol 58: e10 28011869 10.1093/pcp/pcw219

[koac306-B67] Tanigawara M , Tabata KV, Ito Y, Ito J, Watanabe R, Ueno H, Ikeguchi M, Noji H (2012) Role of the DELSEED loop in torque transmission of F1-ATPase. Biophys J 103: 970–978 23009846 10.1016/j.bpj.2012.06.054 PMC3433597

[koac306-B68] Tomizioli M , Lazar C, Brugiere S, Burger T, Salvi D, Gatto L, Moyet L, Breckels LM, Hesse AM, Lilley KS, et al (2014) Deciphering thylakoid sub-compartments using a mass spectrometry-based approach. Mol Cell Proteomics 13: 2147–2167 24872594 10.1074/mcp.M114.040923 PMC4125743

[koac306-B69] Trosch R , Topel M, Flores-Perez U, Jarvis P (2015) Genetic and physical interaction studies reveal functional similarities between ALBINO3 and ALBINO4 in Arabidopsis. Plant Physiol 169: 1292–1306 26265777 10.1104/pp.15.00376 PMC4587442

[koac306-B70] Vollmar M , Schlieper D, Winn M, Büchner C, Groth G (2009) Structure of the c14 rotor ring of the proton translocating chloroplast ATP synthase. J Biol Chem 284: 18228–18235 19423706 10.1074/jbc.M109.006916 PMC2709358

[koac306-B71] von Ballmoos C , Wiedenmann A, Dimroth P (2009) Essentials for ATP synthesis by F1F0 ATP synthases. Annu Rev Biochem 78: 649–672 19489730 10.1146/annurev.biochem.78.081307.104803

[koac306-B72] Yap A , Kindgren P, Colas Des Francs-Small C, Kazama T, Tanz SK, Toriyama K, Small I (2015) AEF1/MPR25 is implicated in RNA editing of plastid atpF and mitochondrial nad5, and also promotes atpF splicing in Arabidopsis and rice. Plant J 81: 661–669 25585673 10.1111/tpj.12756

[koac306-B73] Zhang L , Rochaix JD, Peng L (2020) Regulation of the biogenesis of chloroplast ATP synthase. Adv Bot Res 96: 205–228

[koac306-B74] Zhang L , Duan Z, Zhang J, Peng L (2016) BIOGENESIS FACTOR REQUIRED FOR ATP SYNTHASE 3 facilitates assembly of the chloroplast ATP synthase complex in Arabidopsis. Plant Physiol 17: 00248.02016 10.1104/pp.16.00248 PMC4902607 27208269

[koac306-B75] Zhang L , Zhou W, Che L, Rochaix JD, Lu C, Li W, Peng L (2019) PPR protein BFA2 is essential for the accumulation of the atpH/F transcript in chloroplasts. Front Plant Sci 10: 446 31031784 10.3389/fpls.2019.00446 PMC6474325

[koac306-B76] Zhang L , Pu H, Duan Z, Li Y, Liu B, Zhang Q, Li W, Rochaix J-D, Liu L, Peng L (2018) Nucleus-encoded protein BFA1 promotes efficient assembly of the chloroplast ATP synthase coupling factor 1. Plant Cell 30: 1770–1788 30012777 10.1105/tpc.18.00075 PMC6139693

[koac306-B77] Zoschke R , Kroeger T, Belcher S, Schöttler MA, Barkan A, Schmitz-Linneweber C (2012) The pentatricopeptide repeat-SMR protein ATP4 promotes translation of the chloroplast atpB/E mRNA. Plant J 72: 547–558 22708543 10.1111/j.1365-313X.2012.05081.x

