# Supplementary material for: CGL160-mediated recruitment of the coupling factor CF1 is required for efficient thylakoid ATP synthase assembly, photosynthesis, and chloroplast development in Arabidopsis
Source: Plant Cell. 2022 Oct 17;35(1):488–509. doi: 10.1093/plcell/koac306 (PMC9806626; doi:10.1093/plcell/koac306)
Supplement: koac306_Supplementary_Data [file koac306_supplementary_data.zip › supplement/tpc.22.00301Supplemental Figures and Tables.pdf]

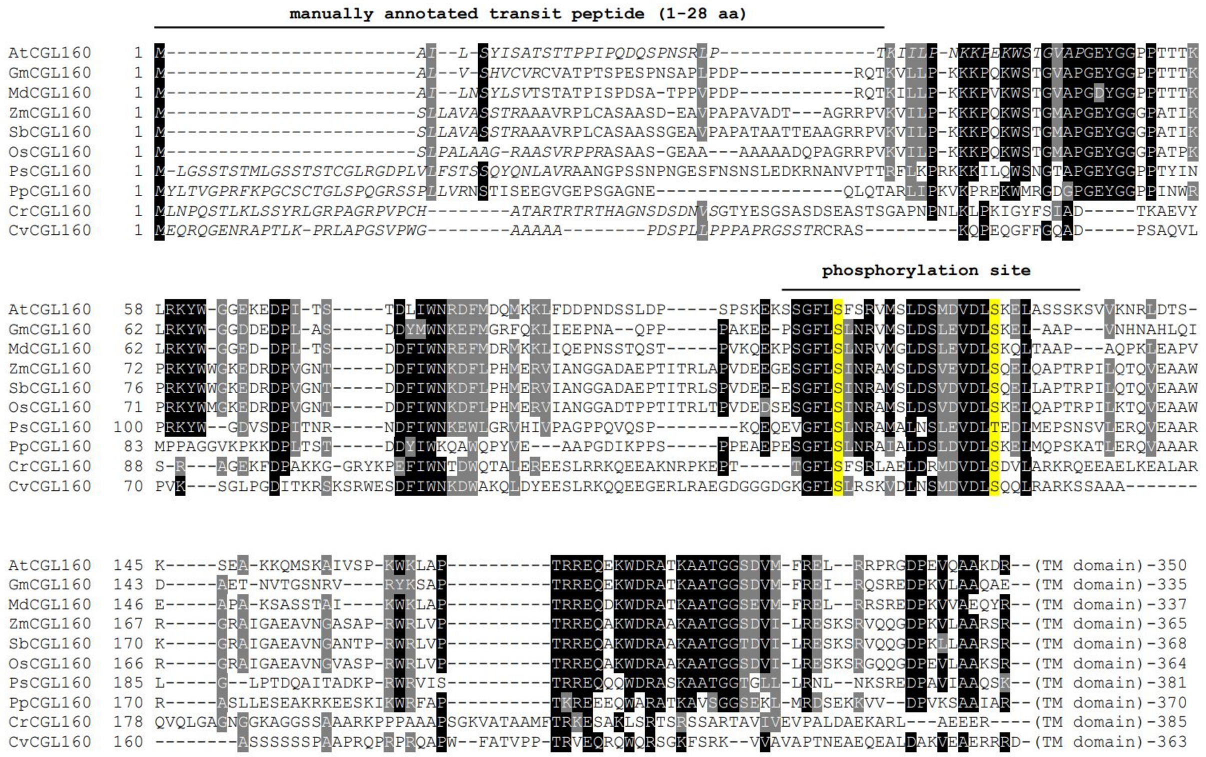

Supplemental Figure S1. **Multiple alignment of the N-terminal portions of CGL160 sequences identified in species belonging to the green lineage.** Putative chloroplast transit peptide (TP) predictions by ChloroP are depicted in italics. The precise length of the AtCGL160 chloroplast TP is unknown and a manual annotation was carried out (1-28 aa) based on conserved amino acids identified in vascular plants. Similar and identical amino acids conserved in 70% of the sequences are highlighted in grey and black, respectively. The region that includes several identified phosphopeptides in AtCGL160 is indicated and two conserved S/T residues are shown in yellow. Note that CGL160 transmembrane (TM) domains were omitted from the alignment. Sequence identifiers for CGL160 homologs are as follows: *Arabidopsis thaliana* (AtCGL160, NP_565711), *Glycine max* (GmCGL160, XP_006582279.1), *Malus domestica* (MdCGL160, XP_008353735.1), *Zea mays* (ZmCGL160, NP_001170362.2), *Sorghum bicolor* (SmCGL160, XP_021312638.1), *Oryza sativa* Japonica group (OsCGL160, XP_015619276.1), *Picea sitchensis* (PsCGL160, ABR16992.1), *Physcomitrella patens* (PpCGL160, XP_024381807.1), *Chlamydomonas reinhardtii* (CrCGL160, XP_001690237.1) and *Chlorella variabilis* (CvCGL160, XP_005844436.1). Supplemental Fig. S1 supports Fig. 1A.

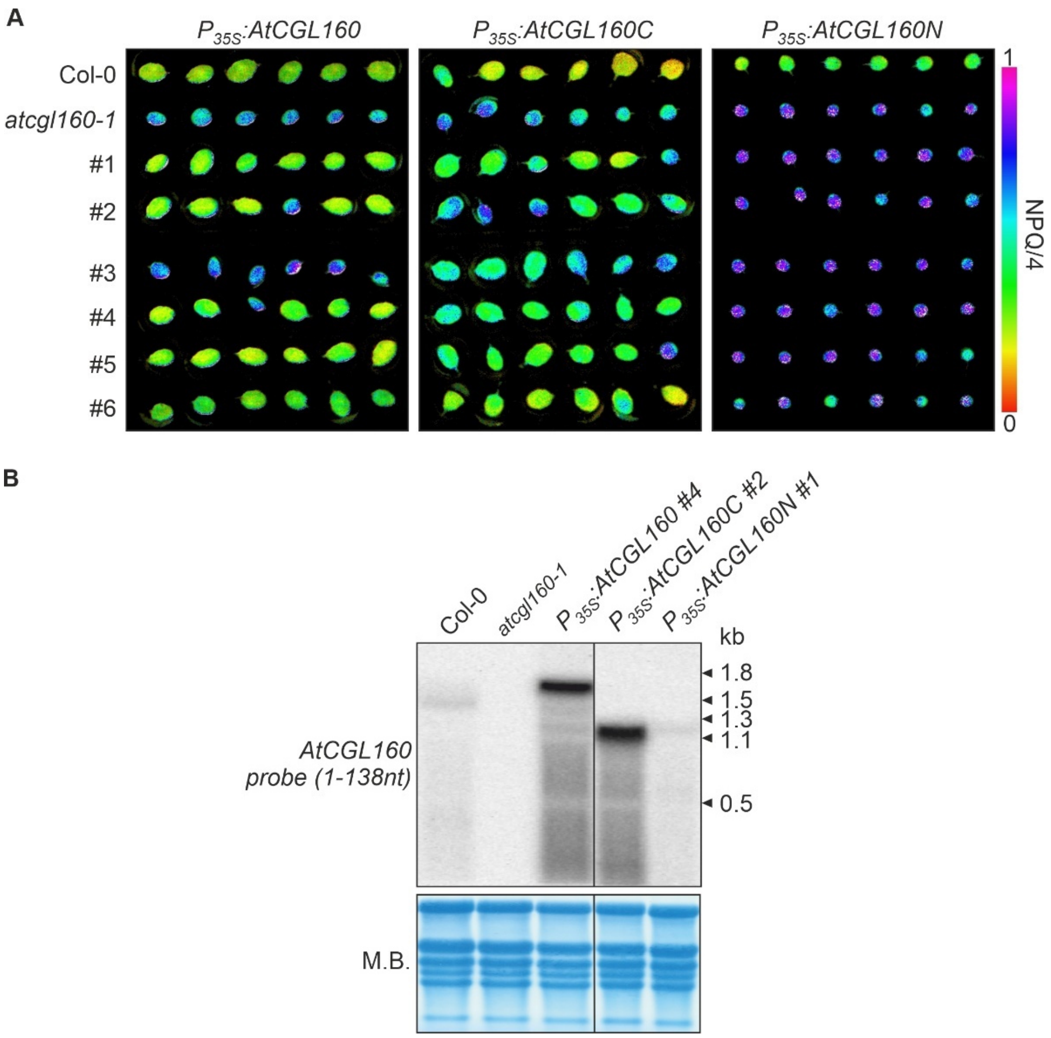

Supplemental Figure S2. Screening of *P₃₅S:AtCGL160, P₃₅S:AtCGL160C*, and *P₃₅S:AtCGL160N* plants. A, After transformation of *atcgl160-1*, T2 offspring of independent T1 plants (#1-#6) were examined using an Imaging-PAM (Walz, Effeltrich, Germany) system. Non-photochemical quenching (NPQ/4) was measured in light induction experiments on detached leaves after 8 min of irradiation at 100 µmol photons m^{-2} s^{-1}, and is indicated on a false-color scale from 0 to 1. Col-0 and *atcgl160-1* leaves served as controls. *P_{35S}:AtCGL160* lines #1, #2, #4, #5 and #6 rescued the *atcgl160-1* phenotype. Transformation of *atcgl160-1* plants with the *P_{35S}:AtCGL160C* and *P_{35S}:AtCGL160N* constructs resulted in partial and no complementation, respectively. **B**, RNA gel blot analyses of selected, homozygous lines (T3 generation). Total RNA (20 µg) from 4-week-old Col-0, *atcgl160-1*, *P_{35S}:AtCGL160, P_{35S}:AtCGL160C and P_{35S}:AtCGL160N* plants was size-fractionated on a denaturing formaldehyde gel and blotted onto a nylon membrane. Hybridization was carried out with a radioactive probe specific for the *AtCGL160* chloroplast transit-peptide coding region (1-138nt) which was present in all constructs. Line #4 (*P_{35S}:AtCGL160)* and line #2 (*P_{35S}:AtCGL160C)* were selected for further experiments due to their similar transcript levels. Methylene blue (M.B.) staining of the nylon membrane served as an RNA loading control. Sizes of rRNAs served as a marker and are indicated in kb. Supplemental Fig. S2 supports Fig. 2.

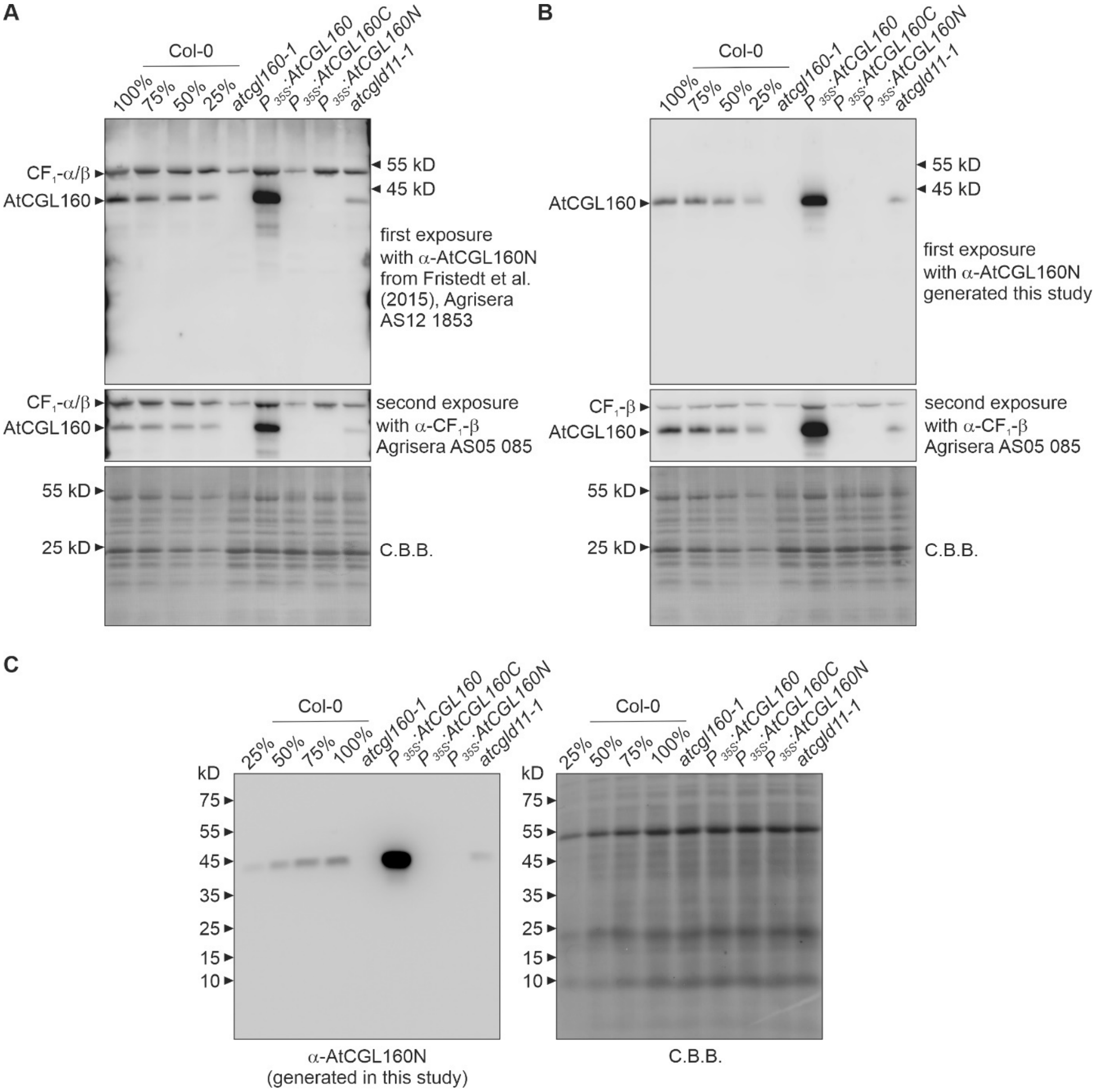

Supplemental Figure S3. Immunodetection of AtCGL160 in Col-0, *atcgl160-1*, *P_{35S}:AtCGL160, P_{35S}:AtCGL160N, P_{35S}:AtCGL160C* and *atcgld11-1* plants. A, Thylakoid proteins were separated by denaturing SDS-PAGE and blotted onto PVDF membranes. Membranes were first probed with an antibody against AtCGL160N (upper panel, AS12 1853, Agrisera). After signal detection, the membrane was re-probed with an antibody against CF_1-β (lower panel, AS05 085, Agrisera). Note that antibody AS12 1853 from Agrisera binds nonspecifically to CF_1-α or CF_1-β (upper panel) and was therefore not considered for use in co-immunoprecipitation, cross-linking or 2D native/SDS-PAGE experiments. **B**, Immunodetection analyses were performed as described in A but using the newly generated antibody against the N-terminal part of AtCGL160. The new antibody did not cross-react with other proteins and was employed for co-immunoprecipitation, cross-linking and 2D native/SDS-PAGE experiments. **C**, AtCGL160 and AtCGL160N amounts in protein extracts isolated from rosette leaves. Protein samples corresponding to 2 mg leaf fresh weight (=100%) were size-fractionated by SDS-PAGE and blotted onto PVDF membranes. AtCGL160 and AtCGL160N were then immunodetected using the newly generated antibody. Coomassie brilliant blue staining (C.B.B.) of PVDF membranes is shown as a loading control. Supplemental Fig. S3 supports Fig. 2.

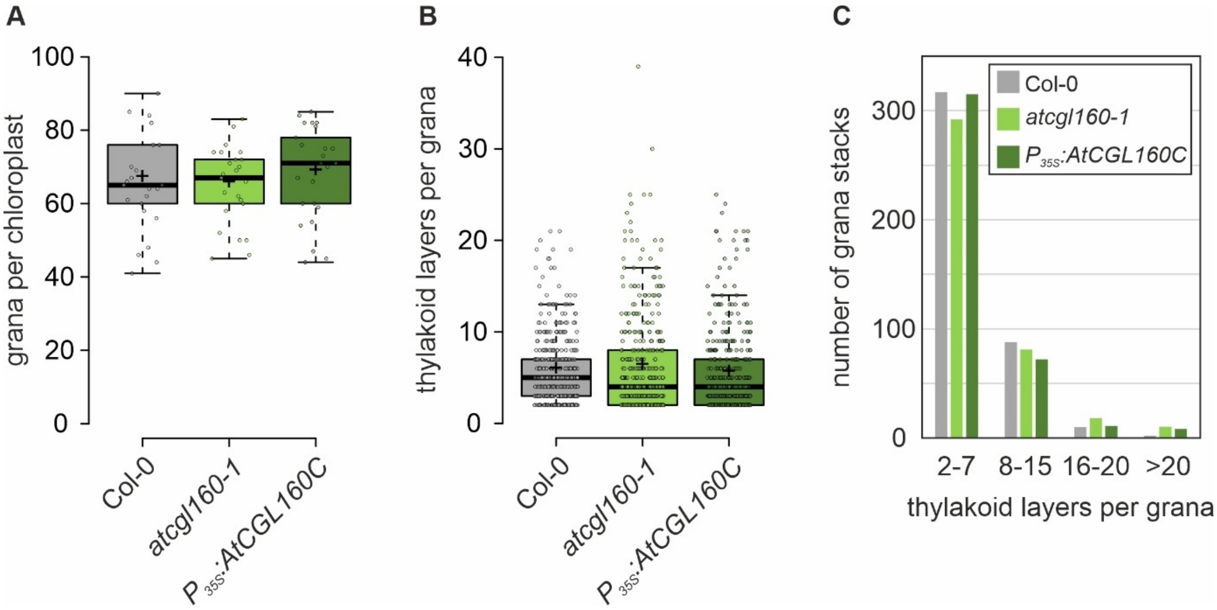

Supplemental Figure S4. Quantification of grana number and height in Col-0, *atcgl160-1* and P_{35S}*:AtCGL160C* green leaf sector samples. A, Grana number (≥2 thylakoid membranes) in 25 chloroplast samples per genotype were counted. The average ± standard deviation for Col-0, *atcgl160-1* and P_{35S}*:AtCGL160C* was 66.8 ± 14.9, 65.24 ± 10.4 and 68.5 ± 12.5, respectively. **B**, The number of thylakoid layers in individual grana stacks was determined from 25 chloroplast samples per genotype. In the case of Col-0, *atcgl160-1* and P_{35S}*:AtCGL160C*, 417, 402 and 406 grana were analyzed, respectively. The average ± standard deviation for Col-0, *atcgl160-1* and P_{35S}*:AtCGL160C* was 5.8 ± 3.8, 6.2 ± 5.4, and 5.4 ± 4.5, respectively. **C**, Distribution of grana stack height in 25 chloroplast samples per genotype. Small (2-7 thylakoid layers), medium (8-15 thylakoid layers), large (16-20 thylakoid layers), and very large grana stacks (>20 thylakoid layers) were grouped according to grana height and plotted against their abundance in the different genotypes. Center lines show the medians. Box limits indicate the 25th and 75th percentiles. Whiskers extend 1.5x the interquartile range from the 25th and 75th percentiles. Outliers are represented by dots. Crosses represent sample means. Data points are plotted as open circles. Col-0, *atcgl160-1* and P_{35S}*:AtCGL160C* data are depicted in grey, light green and dark green colors. Supplemental Fig. S4 supports Fig. 3.

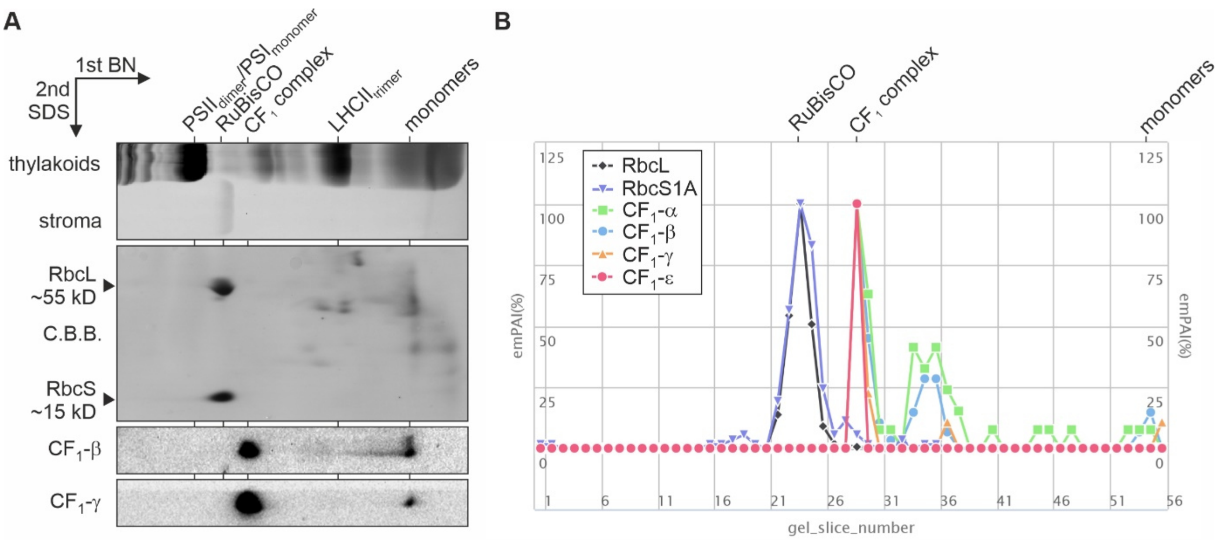

Supplemental Figure S5. Characterization of the stromal CF_1 complex in *atcgl160-1* plants. **A**, A stromal protein extract of *atcgl160-1* plants was subjected to 2D gel electrophoresis (Blue Native- and SDS-PAGE) and immunodetection of CF_1-β and CF_1-γ. Coomassie brilliant blue (G-250) staining of the PVDF membrane after transfer visualized abundant stromal complexes such as RuBisCO, which is composed of RbcL and RbcS. Prominent thylakoid complexes of *P_{35S}:AtCGL160C* plants served as molecular mass standards. **B**, Composition of the stromal CF_1 sub-complex in Arabidopsis according to the Protein Co-migration Database for photosynthetic organisms (PCom-DB, http://pcomdb.lowtem.hokudai.ac.jp/proteins/top). Co-migration of RbcL (black diamonds) and RbcS (purple triangles) is provided for better comparison between PCom-DB results and the 2D gel analyses presented in panel A. Subunit content is quantified according to the exponentially modified protein abundance index (emPAI) method and is normalized for each individual subunit to the maximal emPAI identified in a gel slice (Ishihama et al., 2005). The maximal RuBisCO and CF_1 content were detected in gel slices 24 and 29, respectively. Note that CF_1-δ was not identified in the stromal CF_1 subcomplex. Supplemental Fig. S5 supports Fig. 5.

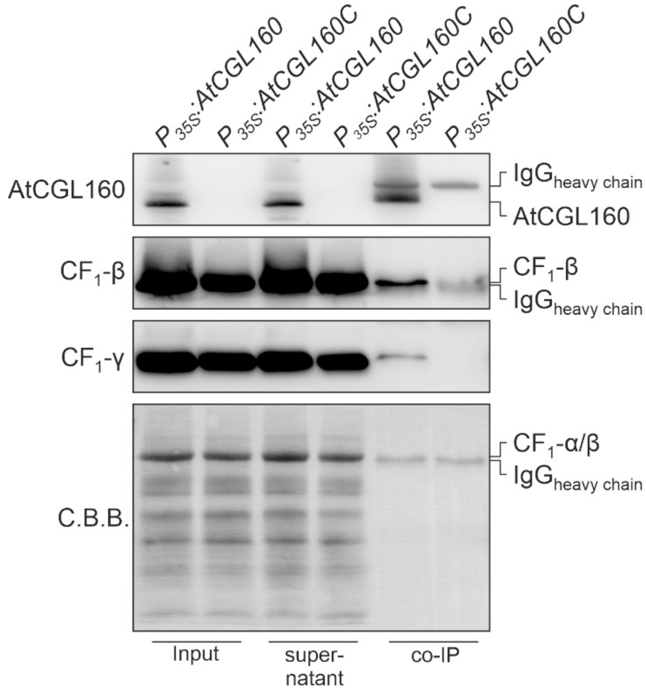

Supplemental Figure S6. Immunoblot analysis of AtCGL160 co-immunoprecipitation assays. Co-immunoprecipitation with NP40-solubilized thylakoids of *P_{35S}:AtCGL160* and *P_{35S}:AtCGL160C* plants was repeated using reduced amounts of the AtCGL160 antibody. Protein A-coupled magnetic beads (Dynabeads, Thermo) coated with AtCGL160 antibody (generated this study) and co-immunoprecipitated proteins (IP) were boiled in SDS loading buffer, separated by denaturing SDS-PAGE and blotted onto PVDF membranes. Samples of NP40-solubilized thylakoids before (Input) and after (supernatant) incubation with AtCGL160 antibody were loaded as controls. Membranes were probed separately with antibodies against AtCGL160N and CF_1-β/CF_1-γ. The positions of the heavy chain of the AtCGL160 antibody are indicated (IgG). Coomassie brilliant blue staining (C.B.B.) is shown as loading control, and the positions of CF_1-α/β are indicated. Supplemental Fig. S6 supports Fig. 6A.

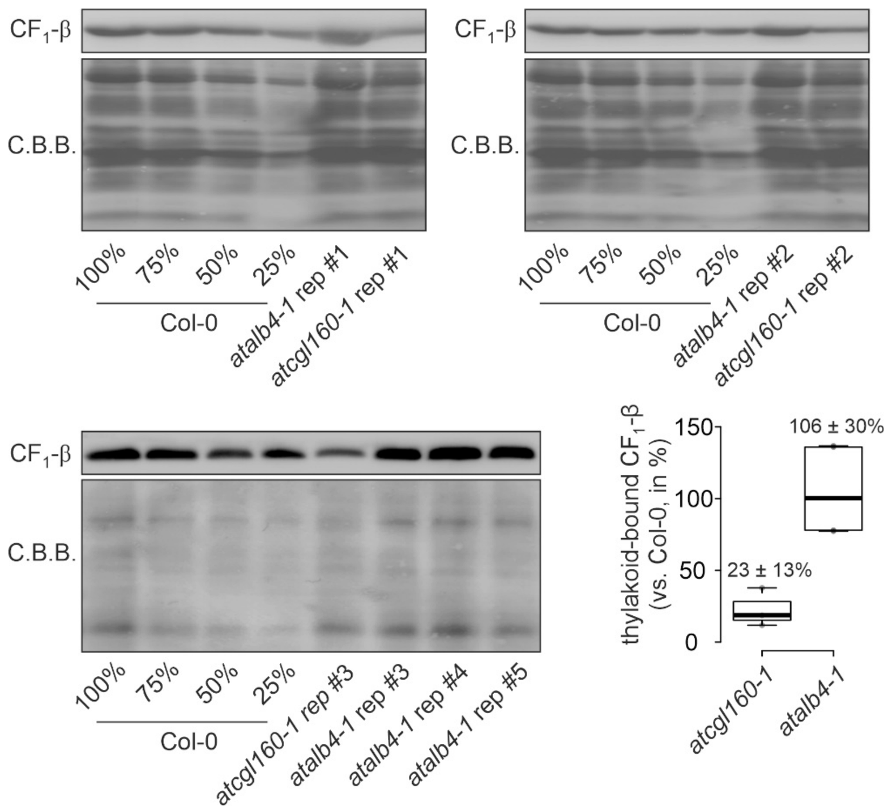

Supplemental Figure S7. Quantification of thylakoid-bound CF$_1$-β subunits in *atalb4-1* Arabidopsis mutant lines. Thylakoid proteins were isolated from Col-0, *atcgl160-1* and *atalb4-1* (SALK_136199C) plants grown under short-day conditions, fractionated on SDS-PAGE, transferred to PVDF membranes and probed with CF$_1$-β antibodies. Membranes were stained with Coomassie brilliant blue G-250 (C.B.B.) as loading control. Signals were quantified relative to signals detected in the wild-type sample using the Bio-1D software (version 15.03, Vilber Lourmat, Eberhardzell, Germany) and are provided as percentages. Horizontal lines represent the median, and boxes indicate the 25th and 75th percentiles. Whiskers extend the interquartile range by a factor of 1.5×. Means ± standard deviations are provided above the boxes. Quantification is based on three and five replicates (rep) for *atcgl160-1* and *atalb4-1* samples, respectively. Supplemental Fig. S7 supports Fig. 6A.

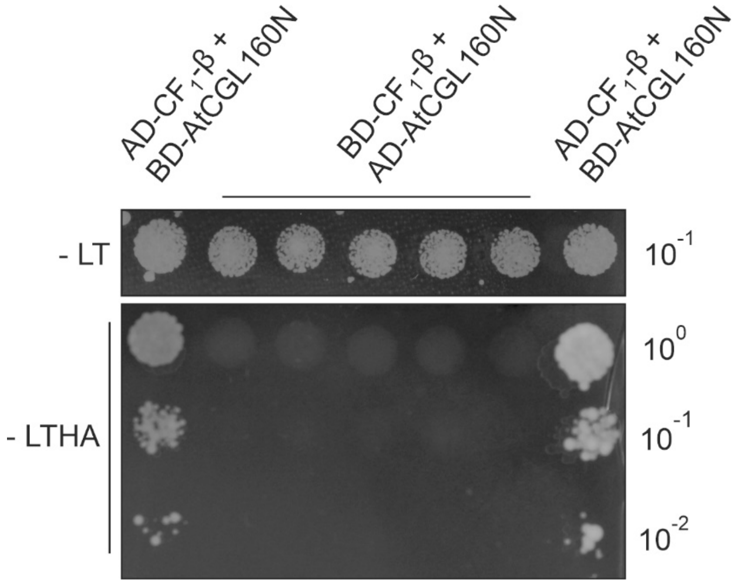

Supplemental Figure S8. Interaction of BD-CF$_1$-β and AD-AtCGL160N in yeast two-hybrid assays. Transformations were verified by plating on permissive medium lacking Leu and Trp (-LT). Then, interactions were tested on selective medium (-Leu/-Trp/-His/-Ade, [-LTHA]) by plating equal numbers of yeast cells in serial dilutions (10^0, 10^{-1}, and 10^{-2}). Co-transformants of AD-CF$_1$-β and BD-AtCGL160N served as positive control (two co-transformants were tested). No interaction of the reciprocal constructs BD-CF$_1$-β and AD-AtCGL160N could be observed (five yeast transformants were tested). Exchanging BD- and AD-fusions could cause changes in the conformation of the bait or prey and lead to altered binding capacity due to steric constraints (Van Criekinge and Beyaert, 1999). Supplemental Fig. S8 supports Fig. 7.

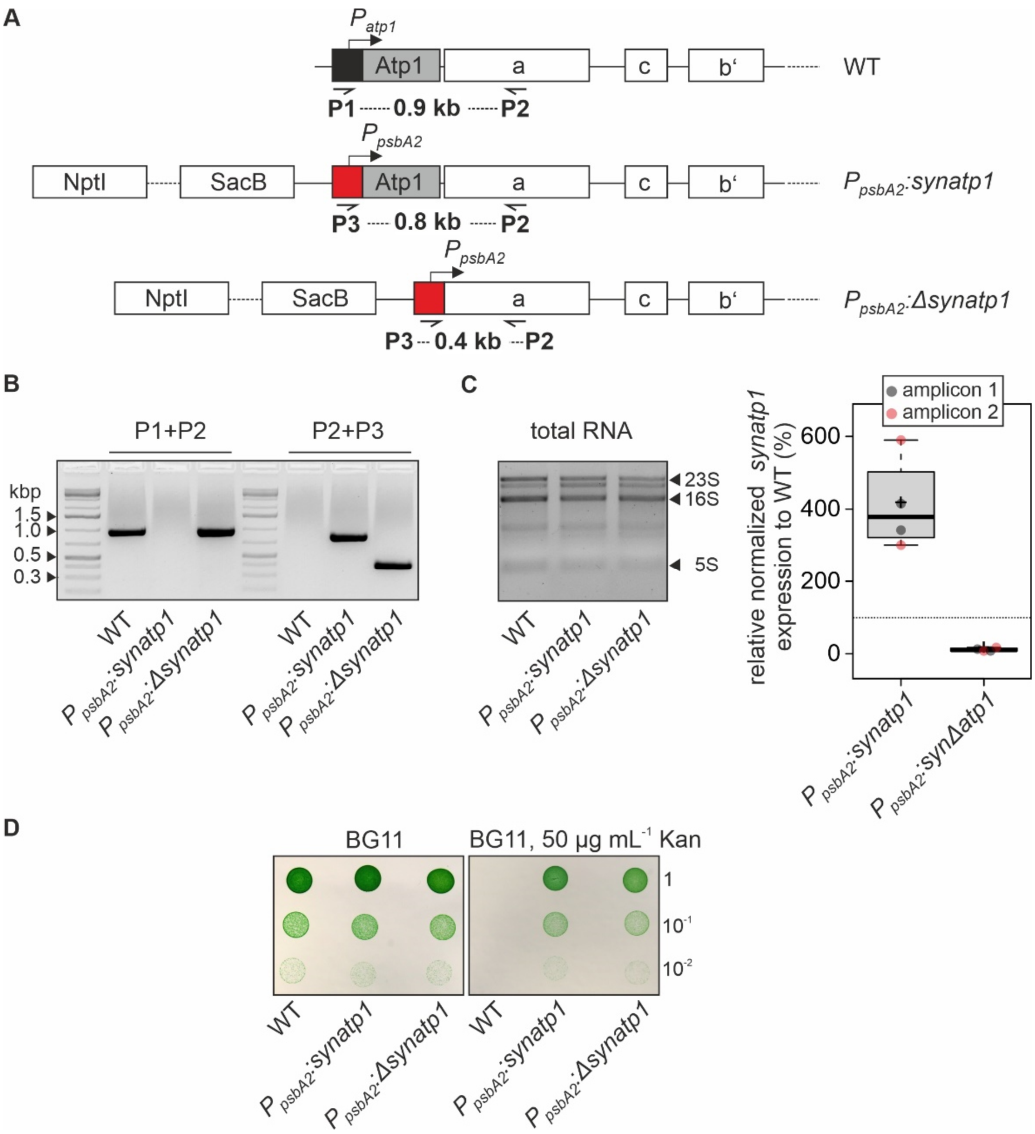

Supplemental Figure S9. Lack of segregation in a *synatp1* knockout strain of *Synechocystis* sp. PCC 6803 (Synechocystis) indicated an essential function for SynAtp1. A, Overview of the *atp1* operon in Synechocystis which is under control of the native *atp1* promoter (P_{atp1}). Atp1 is encoded by the first gene in the operon followed by genes coding for the structural subunits CF_O-a, CF_O-c and CF_O-b'. Subsequent genes coding for CF_O-b, CF_1-δ, CF_1-α and CF_1-γ are not shown. The endogenous promoter P_{atp1} was replaced by the strong *psbA2* promoter P_{psbA2} (indicated in red) in the control strain P_{psbA2}:*synatp1*, whereas P_{psbA2} was placed adjacent to the *atpI* gene (coding for CFo-a) in the P_{psbA2}:*Δsynatp1* strain. A double selection cassette for kanamycin resistance- and sucrose sensitivity-mediating genes *nptI* and *sacB* was employed for selection of positive transformants. **B**, Segregation of P_{psbA2}:*synatp1* and P_{psbA2}:*Δsynatp1* was examined by PCR with primer pairs and product lengths indicated in A. **C**, Expression analysis of *synatp1* in WT, P_{psbA2}:*synatp1* and P_{psbA2}:*Δsynatp1*. Total RNA was isolated and its integrity was examined by gel electrophoresis (left panel). Abundant ribosomal RNAs (5S, 16S and 23S) are labeled. The *synatp1* transcript level was determined by real-time quantitative PCR (RT-qPCR) using two different *synatp1* amplicons. Expression of *synatp1* was normalized to *rrn16S* expression and referred to *synatp1* expression levels of the wildtype (= 100%, dashed line). Center lines show the medians. Box limits indicate the 25th and 75th percentiles. Whiskers extend 1.5 times the interquartile range from the 25th and 75th percentiles. Outliers are represented by dots. Crosses represent sample means and data points are plotted as circles. Data of two RT-qPCR assays with two different *synatp1* amplicons (n = 4 sample points) are shown. **D,** Growth test on BG11 medium. Cells (10 µL of an OD_{730}=0.05 corresponds to a dilution factor of 1) were spread on agar plates in two dilutions (10^{-1} and 10^{-2}) optionally in the presence of 50 µg mL^{-1} kanamycin and exposed to ~60 µmol photons m^{-2} s^{-1} for 96h at 30°C. Supplemental Fig. S9 supports Fig. 8.

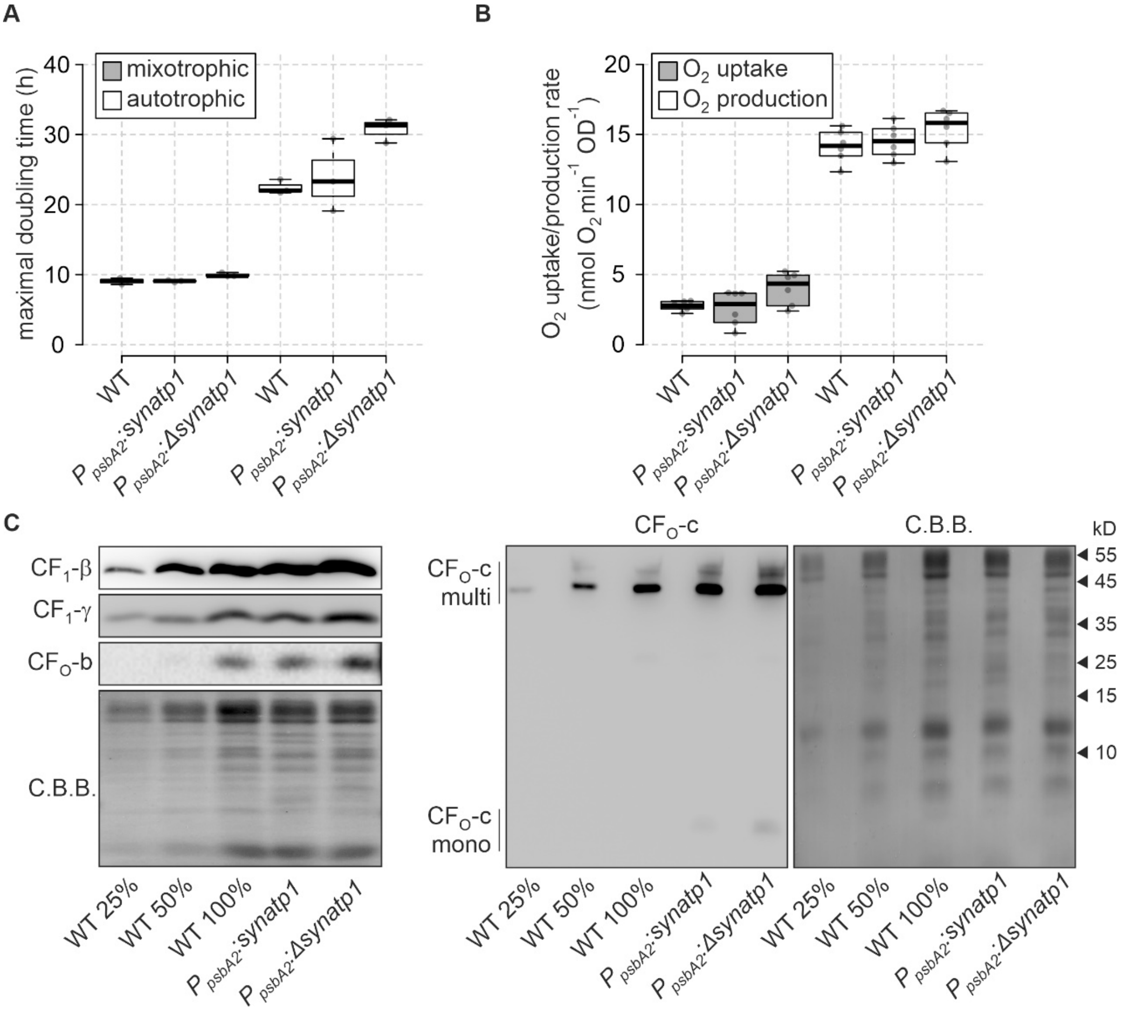

Supplemental Figure S10. The endogenous promoter of the *atp1* operon in Synechocystis could be functionally replaced by the strong *psbA2* promoter (P_{psbA2}). A, Determination of maximal doubling time (in h) under autotrophic and mixotrophic (+ 5 mM glucose) conditions. Cells were inoculated (OD_{730}=0.05) and shaken (120 rpm) in autotrophic or mixotrophic medium for 5 days. Maximal doubling rates were calculated based on daily measured OD_{730} values of three biological replicates per genotype. A biological replicate represented a cell culture inoculated from an independent starter culture. **B,** Oxygen uptake and production rates (nmol O_2 min^{-1} OD_{730}^{-1}) were measured with a Clark-type oxygen electrode (Hansatech Instruments, UK) at 30°C. Oxygen uptake was determined in the dark and production rates at a light intensity of 400 µmol photons m^{-2} s^{-1}. The analysis was conducted with three biological and two technical replications. A biological replicate represented a cell culture inoculated from an independent starter culture. **C,** Immunodetection of CF_1-CF_O marker subunits and CF_O-c multimers (labelled as CF_O-c multi in the right panel). Note that c-rings are stable in the presence of strong detergents such as SDS. Thylakoid samples of wildtype, P_{psbA2}*:synatp1* and P_{psbA2}*:Δsynatp1* corresponding to 2 µg Chl *a* (=100%) were separated by Tricine-SDS-PAGE (10%), transferred to PVDF membranes and decorated with specific antibodies for CF_1-CF_O marker subunits. Membranes were stained with Coomassie brilliant blue G-250 (C.B.B.) to control equal loading. Horizontal lines in boxplots shown in A and B represent the median, boxes indicate the 25th and 75th percentiles and whiskers extend the interquartile range by a factor of 1.5×. Supplemental Fig. S10 supports Fig. 8.

Supplemental Table S1. AtCGL160 co-immunoprecipitation experiments. Differential enriched proteins in *P_{35S}:AtCGL160* versus *P_{35S}:AtCGL160C* samples sorted by $\log_2$ fold change ($-\log_{10}$ *p*-value > 1.5). Nucleus-encoded genes are written in capital letters.

Protein IDs (UNIPROT)	Gene names	$\log_2$ FC	$-\mathrm{Log}_{10}$ *p*-value	adj *p*-value
Q82279	*AtCGL160*	6.495	4.644	0.002
P09468	*atpE*	6.380	2.301	0.035
Q42139	*ATPG*	5.231	4.747	0.002
P56760	*atpH*	4.886	3.176	0.011
Q9SSS9	*ATPD*	4.672	4.523	0.003
P19366	*atpB*	4.437	4.459	0.003
P56759	*atpF*	4.399	5.913	0.000
P56757	*atpA*	4.235	6.410	0.000
Q01908	*ATPC1*	4.156	4.772	0.002
Q2HIU0	*At3g15110*	3.333	3.508	0.007
P56758	*atpI*	2.799	4.104	0.003
O49445	*LECRK72*	2.346	3.115	0.011
Q67XC4	*TBL40*	2.268	2.402	0.031
Q8LCQ4	*LHCA6*	2.082	3.710	0.005
Q39099;A0A1P8B288	*XTH4*	1.983	4.126	0.003
Q41963	*TIP1-2*	1.907	2.821	0.017
O22957	*At2g34040*	1.632	2.829	0.017
F4J519;Q9M9X0;F4J8G2;Q9SRL7;Q9S9U3	*RLP34*	1.564	3.162	0.011
P38418;A0A1I9LPH1	*LOX2*	1.544	2.047	0.049
Q8LBV4	*At1g78140*	1.515	2.171	0.042
F4IUJ0;F4IUI9	*At2g26340*	1.451	3.021	0.013
Q9SRL2;Q9M9X0;F4J8G2;Q9SRL7;Q9S9U3	*RPL35A;RPL35C*	1.254	2.619	0.024
Q9SUI4;A0A1P8B6D0	*PSAL*	1.193	2.938	0.014
Q9FFW9;F4KBJ3	*At5g38520*	1.136	3.084	0.012
Q96242	*CYP74A*	1.078	2.371	0.032
Q9SYW8;F4K8I1	*Lhca2*	0.941	2.356	0.032
Q9SR92	*STR10*	0.839	2.913	0.015
P56777	*psbB*	0.807	2.072	0.048
Q9LHA6	*At3g28220*	0.731	2.315	0.034
Q9S7N7	*PSAG*	0.575	2.157	0.043

Supplemental Table S2. Primers used in this study.

Primer name	Primer sequence 5' to 3'	Comment
oeAtCGL160_s	GGGGACAAGTTTGTACAAAAAAGCAGGCTCAATGGCGATTCTTAGTTACAT	Gateway primer, *P_{35S}:AtCGL160*
oeAtCGL160_as	GGGGACCACTTTGTACAAGAAAGCTGGGTTTAATCACTGGCCTGTGTGT	Gateway primer, *P_{35S}:AtCGL160*
GW_GC9_N_as	GGGGACCACTTTGTACAAGAAAGCTGGGTTTACCTGTCTTTAGCAGCTTGTA	Gateway primer, *P_{35S}:AtCGL160N*
GW_GC9_M_as	GGGGACCACTTTGTACAAGAAAGCTGGGTTTAATCACTGGCCTGTGTGT	Gateway primer, *P_{35S}:AtCGL160C*
TP-GC9C_fus_s	GGTCCACCGGAGTTGCTCCCGAACAATATTTTAAGCTGAA	Fusion PCR, *P_{35S}:AtCGL160C*
TP-Gc9C_fus_as	TTCAGCTTAAAATATTGTTCGGGAGCAACTCCGGTGGACC	Fusion PCR, *P_{35S}:AtCGL160C*
AtCGL160N_SynAtp1_fus_s	TACAAGCTGCTAAAGACAGGGCAGACTTCTACAGGCTGCA	Fusion PCR, *P_{35S}:AtCGL160N-SynAtp1*
AtCGL160N_SynAtp1_fus_as	TGCAGCCTGTAGAAGTCTGCCCTGTCTTTAGCAGCTTGTA	Fusion PCR, *P_{35S}:AtCGL160N-SynAtp1*
GW_SynAtp1_as	GGGGACCACTTTGTACAAGAAAGCTGGGTTCAATCAGCGGCCGGCGTAA	Gateway primer, *P_{35S}:AtCGL160N-SynAtp1*
CGL160-MBP_s	AAAATCATTCTACCCAATAA	MBP cloning primer
CGL160N-MBP_as	GGTCCTGAATTCTTACCTGTCTTTAGCAGCTTGTA	MBP cloning primer
GST-CGL160N-s	GGGGACAAGTTTGTACAAAAAAGCAGGCTCAAAAATCATTCTACCCAATAAGAAACCTGA	Gateway primer
GST-CGL160N-as	GGGGACCACTTTGTACAAGAAAGCTGGGTCTTACCTGTCTTTAGCAGCTTGTAC	Gateway primer
cgl160cTP_probe_s	ATGGCGATTCTTAGTTACATCTCAGC	Northern probe
cgl160cTP_probe_as	GGGAGCAACTCCGGTG	Northern probe
pGBKT7-CGL160N_s	GGTGGTCATATGAAAATCATTCTACCCAATAAGA	Y2H cloning primer
pGBKT7-CGL160N_as	GGTCCTGAATTCTTACCTGTCTTTAGCAGCTTGTA	Y2H cloning primer
pGADT7-alpha_s	GGTGGTCATATGGTAACCATTAGAGCCGACGA	Y2H cloning primer
pGADT7-alpha_as	GGTCCTGAATTCTTATACTTTCTCCTGAAGTA	Y2H cloning primer
pGADT7-beta_s	GGTGGTCATATGAGAACAAATCCTACTACTTC	Y2H cloning primer
pGADT7-beta_as	GGTCCTGAATTCTCATTTCTTCAATTTACTCT	Y2H cloning primer
pGADT7-gamma_s	GGTGGTCATATGGCTTCCTCTGTTTCACCACT	Y2H cloning primer
pGADT7-gamma_as	GGTCCTGAATTCTCAAACCTGTGCATTAGCTC	Y2H cloning primer
pGADT7-delta_s	GGTGGTCATATGGCCACCGCAGCATCAAGCTA	Y2H cloning primer
pGADT7-delta_as	GGTCCTGAATTCTCAAGTAGCTAATTGAATCT	Y2H cloning primer
pGADT7-epsilon_s	GGTGGTCATATGACCTTAAATCTTTGTGTACTGACTC	Y2H cloning primer
pGADT7-epsilon_as	GGTCCTGAATTCTCAAATCGTATTGAGAGCCT	Y2H cloning primer
pGADT7-AtCGL160_s	GGTGGTCATATGAAAATCATTCTACCCAATAAGA	Y2H cloning primer
pGADT7-AtCGL160_as	GGTCCTGAATTCTTACCTGTCTTTAGCAGCTTGTA	Y2H cloning primer
pGADT7-AtCGLD11_s	GGTGGTCATATGTCTTCGAGTCTATGGAAGCT	Y2H cloning primer
pGADT7-AtCGLD11_as	GGTCCTGAATTCTTAACCCTGGAGTAATTTCA	Y2H cloning primer
pGADT7-atpFsoluble_s	GGTGGTCATATGGATTTATTAGATAACCGAAAG	Y2H cloning primer
pGADT7-atpFsoluble_as	GGTCCTGAATTCTTAATCAGTTATTTCTTTCATCG	Y2H cloning primer
pGADT7-atpGsoluble_s	GGTGGTCATATGCCGCTTGGTAACTTCATGG	Y2H cloning primer
pGADT7-atpGsoluble_as	GGTCCTGAATTCTTAAGAAGGAAGAACCTTCTTGAC	Y2H cloning primer
pGADT7_AtpBI-AD_s	CGCGAATTCATGAGAACAAATCCTAC	Y2H cloning primer
pGADT7_AtpBI-AD_as	ACTCTCGAGTCAATTTCCCATATCAACCAC	Y2H cloning primer

pGADT7_AtpBII-AD_s	ATGGAATTCCCTCTAAGTGTTCCAG	Y2H cloning primer
pGADT7_AtpBII-AD_as	AACCTCGAGTCAAGGTTGTAGCATAGTTG	Y2H cloning primer
pGADT7_AtpBIII-AD_s	CTAGAATTCCGAATCGTTGGCGAG	Y2H cloning primer
pGADT7_AtpBIII-AD_as	GCGCTCGAGTCATTTCTTCAATTTACTC	Y2H cloning primer
pGBKT7_CGL160N_del29_73_s	GACTTAATCTGGAACAGAGATTTTATGG	Y2H cloning primer
pGBKT7_CGL160N_del29_73_as	CATATGCAGGTCCTCCTCT	Y2H cloning primer
pGBKT7_CGL160N_del74_105_s	GTCTTCTGGGTTTCTGAG	Y2H cloning primer
pGBKT7_CGL160N_del74_105_as	GTGGAAGTAATGGGATCTTC	Y2H cloning primer
pGBKT7_CGL160N_del106_134_s	CGTTGTGAAAAATCGTCTTGACAC	Y2H cloning primer
pGBKT7_CGL160N_del106_134_as	GACTTTTCCTTTGAAGGAGATGG	Y2H cloning primer
pGBKT7_CGL160N_del135_160_s	GAAGCTGGCACCTACACG	Y2H cloning primer
pGBKT7_CGL160N_del135_160_as	CATTTAGAAGACGATGCAAGCTCTTTACTTAAATC	Y2H cloning primer
pGBKT7_CGL160N_del161_206_s	GAATTCCCGGGGATCCG	Y2H cloning primer
pGBKT7_CGL160N_del161_206_as	CTATTTAGGAGACACAATAGCCTTACTCATTTG	Y2H cloning primer
HR1_for	TTTGGTCTCTAGGTAATCCACTGGTAAATCGTCCGC	P_{psbA2}:*synatp1*, P_{psbA2}:*Δsynatp1*, HR1 box
HR1_rev	TTTGGTCTCTAACGTTGGTAATCAAGGACCACCTACC	P_{psbA2}:*synatp1*, P_{psbA2}:*Δsynatp1,* HR1 box
nptI_sacB_for	TTTGGTCTCTCGTTGGAATTCGATTGATCCGTCGACC	P_{psbA2}:*synatp1*, P_{psbA2}:*Δsynatp1,* double selection cassette
nptI_sacB_rev	TTTGGTCTCTCATACTTTAGGCCCGTAGTCTGCAAAT	P_{psbA2}:*synatp1*, P_{psbA2}:*Δsynatp1,* double selection cassette
P_psbA2_for	TTTGGTCTCTTATGCCCATGGAAAAAACGACAATTAC	P_{psbA2}:*synatp1*, P_{psbA2}:*Δsynatp1*, *psbA2* promoter
P_psbA2_rev	TTTGGTCTCTCCATTTGGTTATAATTCCTTATGT	P_{psbA2}:*synatp1*, *psbA2* promoter
atp1_rev_KO	TTTGGTCTCTGCATTTGGTTATAATTCCTTATGT	P_{psbA2}:*Δsynatp1*, *psbA2* promoter
HR2_for	TTTGGTCTCTATGGCTGACTTTTACCGGTT	P_{psbA2}:*synatp1*, HR2 box
HR2_for_KO	TTTGGTCTCT ATGCAAGGGAGCCTCTCCTG	P_{psbA2}:*Δsynatp1*, *psbA2*
HR2_rev	TTTGGTCTCTAAGCATGGCCAGGTTAAAGAGGTTGG	P_{psbA2}:*synatp1*, P_{psbA2}:*Δsynatp1*, HR2 box
P1 (atp1_for)	GGGTAGGTGGTCCTTGATTA	P_{psbA2}:*synatp1* genotyping
P2 (synatpi_rev)	GTATTCCTTTTCCCCCAA	P_{psbA2}:*synatp1*, P_{psbA2}:*Δsynatp1* genotyping
P3 (psbA2_v2_for)	CCAATCTGAACATCGACA	P_{psbA2}:*Δsynatp1* genotyping
qPCR_synatp1_for	CAACGGCAGTTGTTGACCTG	*synatp1* amplicon 1, qPCR
qPCR_synatp1_rev	GGCGTAAACACGCTTTGCAG	*synatp1* amplicon 1, qPCR
qPCR_synatp1_V2_for	GTTGACCTGGACCCTGGTAG	*synatp1* amplicon 2, qPCR
qPCR_synatp1_V2_rev	CCCTTTGGGTGGCAATGATG	*synatp1* amplicon 2, qPCR
BD16SF1	CACACTGGGACTGAGACAC	*rrn16S,* qPCR
BD16SR1	CTGCTGGCACGGAGTTAG	*rrn16S,* qPCR
