# Supplementary material for: CGL160-mediated recruitment of the coupling factor CF1 is required for efficient thylakoid ATP synthase assembly, photosynthesis, and chloroplast development in Arabidopsis
Source: Plant Cell. 2022 Oct 17;35(1):488–509. doi: 10.1093/plcell/koac306 (PMC9806626; doi:10.1093/plcell/koac306)
Supplement: koac306_Supplementary_Data [file tpc22.00301.PeerReviewReport.pdf › supplement/tpc22.00301.PeerReviewReport.pdf]

CGL160-mediated recruitment of the coupling factor CF_1 is required for efficient thylakoid ATP synthase assembly, photosynthesis, and chloroplast development in Arabidopsis

Bennet Reiter, Lea Rosenhammer, Giada Marino, Stefan Geimer, Dario Leister, Thilo Rühle

Corresponding author: Thilo Rühle, thilo.ruehle@biologie.uni-muenchen.de

Review timeline:

**TPC2020-RA-00301**	Submission received:	Mar. 27, 2022
	1st Decision:	June 7, 2022 *revision requested*
**TPC2020-RA-00301R1**	1st Revision received:	June 22, 2022
	2nd Decision:	Sept. 25, 2022 *acceptance pending, sent to science editor*
	Final acceptance:	Oct. 11, 2022

REPORT: (The report shows the major requests for revision and author responses. Minor comments for revision and miscellaneous correspondence are not included. The original format may not be reflected in this compilation, but the reviewer comments and author responses are not edited, except to correct minor typographical or spelling errors that could be a source of ambiguity.)

TPC2020-RA-00301 1st Editorial decision – *revision requested* **June 7, 2022**

We have received reviews of your manuscript entitled "AtCGL160 recruits chloroplast coupling factor 1." Thank you for submitting your best work to The Plant Cell. The editorial board agrees that the work you describe is substantive, falls within the scope of the journal, and may become acceptable for publication, pending revision and potential re-review.

While we ask you to pay careful attention to all of the reviewers' criticisms while preparing your revision, the following points are especially important:

1) Clarify the use of antibodies (see Reviewer #1 comment 1). Please also see the comment about Supp Fig. 3 by Reviewer #2.

2) Address the concerns of Reviewer #2 about Supp Fig. 2.

3) Data on Atp1 expression in the PpsbA2:synatp1 Synechocystis strain should be provided (see major comment #1 by Reviewer #3).

4) The authors should consider revising the title and abstract to address the concerns of Reviewer #3 (last two major points).

-- Reviewer comments:

[Reviewer comments shown below along with author responses]

TPC2020-RA-00301R1 1st Revision received **June 22, 2022**

Reviewer comments and **author responses**:

Editor:

1) Clarify the use of antibodies (see reviewer #1 comment 1). Please also see the comment about Supp Fig. 3 by reviewer #2.

> **Response: We now state more clearly in the revised manuscript when the old or new antibody were used. In addition, the paragraph on the generation of the antibody has been moved to the beginning of the results section.**

2) Address the concerns of reviewer #2 about Supp Fig. 2.

Response: Supplemental Fig. 2 has been revised in the new manuscript. For more details, see our response to reviewer #2 concerning Supplemental Fig. 2.

3) Data on *Atp1* expression in the PpsbA2:synatp1 Synechocystis strain should be provided (see major comment #1 by reviewer #3).

Response: Data on synatp1 expression are now provided (See our response to major comment #1 by reviewer #3 in the section below).

4) The authors should consider revising the title and abstract to address the concerns of reviewer #3 (last two major points).

Response: We addressed the last two major points of reviewer #3 (see section below for more details).

Reviewer #1:

The authors are investigating the role of AtCGL160 in the assembly of the CF1-CFO ATP synthase of chloroplast thylakoid in Arabidopsis. AtCGL160 is a transmembrane thylakoid protein with a fairly large soluble domain, which the authors demonstrate is in the stroma. Previous studies demonstrated the involvement of AtCGL160 in c-ring assembly of the CFO portion and strongly suggest through crosslinking and immunodetection a possible involvement of the soluble portion in integration of the CF1 into the holocomplex. The current study does a really nice job of demonstrating the involvement of the soluble domain of AtCGL160 in interacting with the CF1 portion of the thylakoid ATP synthase, specifically through interaction with the β subunit in its thylakoid-proximal region. This study provides strong evidence that CGL160 does play a role in coordinating the assembly of CF1 with CFO to form the holocomplex.

Overall, this is a really nice study that was carefully done and the conclusions drawn from the experimental outcomes are sound and logical. I have a few organizational or clarifying minor issues with the manuscript as listed below in order of importance.

The authors use immuno-detection and coIP methods to demonstrate the presence and interaction of AtCGL160, AtCGL160N, etc., with other components. Then after several figures, some of which used immuno-detection, they present that the antibody used in a prior study cross-reacted with the CF1-β (i.e., lines 325-335 and Supplemental Fig. S3). It would be clearer if the authors maybe led with the genetic studies and then focus on the protein with the more biochemical studies (or vice versa) instead of going back and forth between the two approaches. Or when presenting the generation of the new antibody and why in the text, please be very clear about which experiments used the new Ab and which used the old.

Response: We now present the generation of the new AtCGL160 antibody earlier in the manuscript and provide information in which experiment we used the old or the new antibody.

2) Figures 1 and 7: In figure 1 the authors indicate that AtCGL160N is amino acids 47-206 and the predicted signal peptide is 1-46; yet, in figure 7 and the methods for the Y2H, they indicate they used a construct containing aa 29-206 lacking the signal peptide. Please clarify the length of the signal peptide. If the construct was aa 29-206, why did the authors include a portion of the signal peptide in the Y2H construct and then show that it is critical to interaction with CF1 β? Do the authors conclude that it is the region 47-74 responsible for the interaction with CF1 β? Why include the extra amino acids?

Response: We agree with the reviewer that we did not precisely discuss the ambiguity of chloroplast transit peptide (TP) prediction and corrected it in the manuscript. Chloroplast TP prediction was carried out by ChloroP and gave rise to a putative AtCGL160 chloroplast TP of 46aa. However, AtCGL16029- 46 aa is conserved in other vascular plants (Supplemental Fig. 1), indicating incorrect chloroplast TP prediction by ChloroP. Thus, we also considered AtCGL160 (29-46 aa) in Y2H assays. We concluded that cTP prediction by ChloroP might be incorrect and that the 29-46aa region is part of the domain in the mature protein which is responsible for interaction with CF1-β.

We changed the color code in Fig. 1 and write now in the legend: 'The precise length of the AtCGL160 chloroplast transit peptide (TP) is unknown. ChloroP chloroplast TP prediction (1-46 aa) and manual annotation (1-28 aa) based on sequence comparison (Supplemental Fig. 1) is depicted in green and bright green, respectively.' Moreover, we specify in the main text: '…However, the alignment of AtCGL160 and CGL160N sequences from other species in the green lineage (Supplemental Fig. 1) revealed that AtCGL16029- 46 aa is conserved in vascular plants, indicating incorrect chloroplast TP prediction by ChloroP. …'

3) Figure 7: did the reciprocal Y2H constructs (with AtCGL160N on the AD and CF1 β on the BD) show a similar result?

Response: We cloned the BD-CF1 -β Y2H construct and tested for interaction between BD-CF1-β and AD-AtCGL160N. However, no growth of yeast cells on selective -LTHA medium was observable. As discussed in the review of Van Criekinge and Beyaert (1999, Yeast two-hybrid: State of the art. Biol. Proced. Online 2: 1–38), exchanging prey and bait fusions is not trivial, since the BD- or AD-fusions might cause changes in the conformation of the bait or prey and lead to altered binding capacity due to steric constraints.

We provide the new data in Supplemental Fig. 7 and refer to them in the legend of Fig. 7: 'Note that the reciprocal constructs BD-CF1-β and AD-AtCGL160N did not interact in yeast two-hybrid assays (Supplemental Fig. 7).'

We write in the legend of Supplemental Fig. 8 in the revised manuscript:'…No interaction of the reciprocal constructs BD-CF1-β and AD-AtCGL160N could be observed (five yeast transformants were tested). Exchanging BD- and AD-fusions could cause changes in the conformation of the bait or prey and lead to altered binding capacity due to steric constraints (Van Criekinge and Beyaert, 1999)…'

4) Supplemental Fig. S6 seems to be out of sequence. It is introduced for the first time and discussed in the Discussion section after the Results section. It would be more appropriately reordered to reflect this.

Response: We now introduce the Supplemental Figure (now Supplemental Fig. 7) in the results section (after the presentation of the co-IP results) and describe the main findings prior to their discussion. We write: 'Since ALB4, which is a member of the bacterial ALB3/Oxa1/YidC protein insertase family, was previously proposed to participate in the linkage of a CF1 to a CFO assembly module (Benz et al., 2009), but was not pulled down in co-IP assays (Supplemental Table 1), the amount of thylakoid- associated CF1 complexes were re-assessed in atalb4-1 (SALK_136199C) mutants and compared to levels identified in atcgl160-1 plants (Supplemental Fig. 7). Thylakoids were isolated from Col-0, atcgl160-1 and atalb4-1 plants grown under short-day conditions, and subjected to immunodetection assays of subunit CF1-β. CF1-β levels bound to atcgl160-1 thylakoids were reduced to 23 ± 13% wild-type level, while no deviation to the wild-type control could be observed for thylakoid- associated CF1-β content in atalb4-1 samples (106 ± 30%).'

<u>Reviewer #2:</u>

The manuscript "The Arabidopsis Protein CONSERVED ONLY IN THE GREEN LINEAGE160 Promotes the Assembly of the Membranous Part of the Chloroplast ATP Synthase" by Rühle et al. describes a detailed functional characterization of AtCGL160 in chloroplast ATP synthase assembly. The authors' group has already published a paper on the characterization of the function of AtCGL160 in cpATP synthase and concluded that this protein is required for the normal accumulation of cpATP synthase (Plant Physiol, 2015). They reported that AtCGL160 is involved in c-ring assembly and proposed that the N-terminal half of AtCGL160 has a regulatory role in the assembly. The AtCGL160 has a hydrophobic N-terminal domain and a hydrophobic Atp1/UncI-like C-terminal domain. The Atp1/UncI in prokaryotes is involved in the ring assembly of the c-ring in FO. The present study mainly aims to assess the function of the N-terminal domain in the assembly of cpATP synthase. The authors transformed atcgl160-1 with the constructs that express AtCGL160, AtCGL160N, and AtCGL160C under the control of the 35S promoter. Although the P35S:AtCGL160N did not recover the phenotype, P35S:AtCGL160C partially rescued the phenotype. The authors suggested that the AtCGL160C assists the c-ring assembly, but the integration of the CFO and CF1 was not so efficient as that in Col-0. Interaction of AtCGL160 with subunits of cpATP synthase was also assessed by using co- immunoprecipitation analyses, and it was shown that not only c- and b'-subunits in FO but also some subunits in F1 have interactions with AtCGL160. Also, yeast two-hybrid assays showed that beta-subunit in F1

interacts with AtCGL160N, and, in particular, the domain close to the FO has a stronger affinity with AtCGL160N. The authors concluded that the N- terminal domain of CGL160 (AtCGL160N) is required for late steps in CF1-CFO assembly. The experiments were carried out properly, and the data are sound in this manuscript. The conclusion that the AtCGL160 is involved in not only the c-ring assembly but also assembly of CF1-CFO assembly is reasonable. However, the data showing the interaction of AtCGL160 with specific subunits of cpATP synthase are not sufficient for confident evidence that supports the conclusion.

1) It is necessary to show that newly synthesized subunits of cpATP synthase specifically interact with AtCGL160.

Response: We repeated the co-immunoprecipitation experiment (Figure 6) with [^{35}S]Met labeled proteins (Supporting Figure 1 in this document).

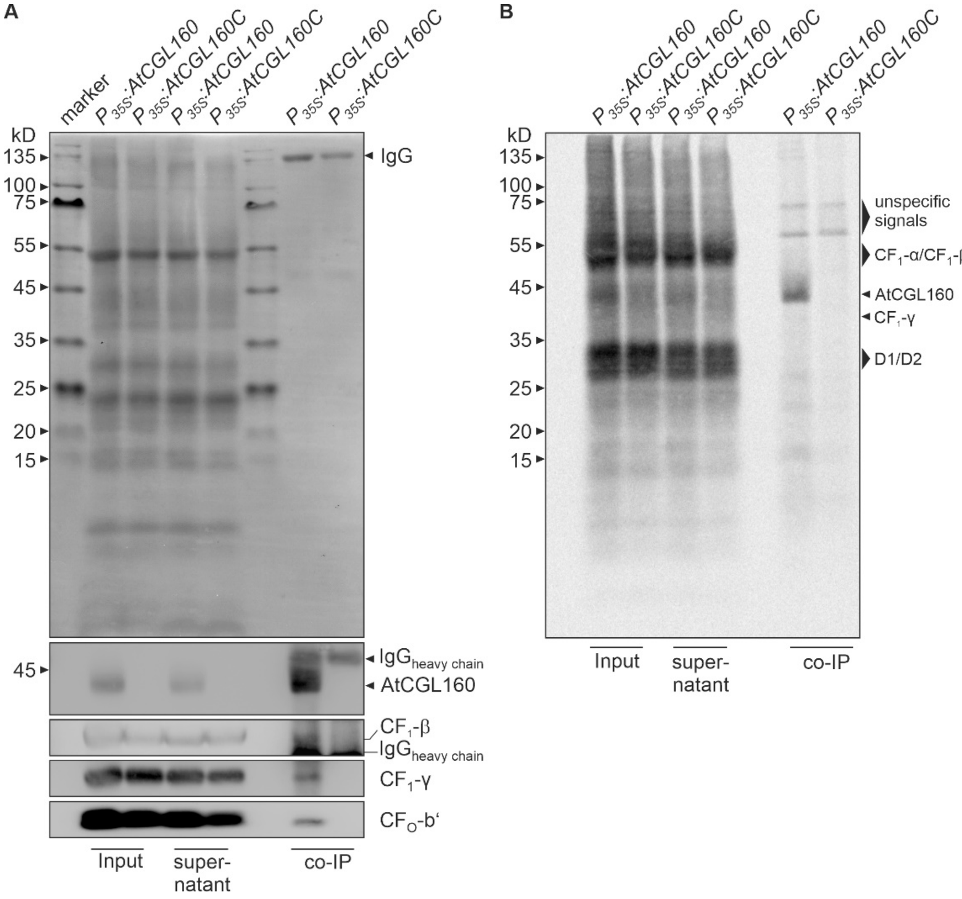

Supporting Figure 1. Co-immunoprecipitation analyses using [35S]Met labeled thylakoid proteins. First, P35 S:AtCGL160 and P35 S:AtCGL160C leaves were infiltrated for 2h with [35S]Met. No cycloheximide was added, since CFO-b', CF1 -δ and CF1 -γ are nucleus-encoded and are required for correct chloroplast ATP synthase assembly. Then, thylakoids were isolated and protein complexes were solubilized by an NP40-containing buffer as described in the manuscript. After co-IP with the newly generated AtCGL160N-specific antibody, pulled-down and labeled proteins were eluted with SDS-PAGE loading buffer from protein A magnetic beads (NEB), size-fractionated by electrophoresis and blotted onto a PVDF membrane. A, Visualization of transferred proteins by Coomassie staining (upper panel). Immunodetection of AtCGL160 (with the newly generated antibody), CF1 -γ and CFO-b' (lower panels). B, Detection of labeled thylakoid proteins using a storage phosphor imager (Typhoon scanner, GE Healthcare). Expected positions of labeled CF1 -α/β/γ are indicated. Note that Supporting Figure 1 is not included in the revised manuscript and only part of the 'Response to reviewer' document. Supporting Figure 1 refers to the response to reviewer #2, comment 1.

We were able to immunoprecipitate labeled AtCGL160 (labeling was carried out without blocking cytosolic translation) and could confirm the pulldown of chloroplast ATP synthase subunits in immunodetection assays.

However, labeled chloroplast ATP synthase subunits were not co-immunoprecipitated from solubilized P35S:AtCGL160 thylakoid extracts to detectable amounts which might be due to the low turnover rate of chloroplast ATP synthase biogenesis and the transient character of the AtCGL160/CF1-β interaction as demonstrated by our cross-linking experiments (Fig. 6C). We do not want to include the results in the manuscript, since in our opinion the data (co-IP with unlabeled thylakoid proteins, cross-linking/co-migration and yeast two-hybrid experiments) are sufficient to state an association of AtCGL160 with CF1-containing complexes.

2) Supplemental Figure 2: Two Northern blot analyses in panels B and C are unclear to me. What is the difference between the results in panels B and C? Why was no signal detected in Col-0 in panel B? Why there are two #2 lanes. P35S:AtCGL160C? In the legend, line #1 (P35S:AtCGL160) may be #4. In L178, the authors describe that AtCGL160N transcripts were in WT-like amounts present in P35S:AtCGL160N plants. The size markers should be indicated.

Response: We agree with the reviewer that the first Northern blot analysis shown in Supplemental Fig. 2 in the manuscript was of low quality (e.g. too low amounts of RNA were loaded to detect AtCGL160 transcripts in Col-0) and sample loading was confusing. Thus, we rearranged Supplemental Fig. 2 and deleted the results of the first Northern blot analysis (Supplemental Fig. 2B in the former manuscript). It should now be more obvious that expression levels of AtCGL160N detected in P35S:AtCGL160N #1 plants were close to AtCGL160 expression levels identified in Col-0. We also changed the order of genotypes in the Imaging-PAM analyses (we exchanged the P35S:AtCGL160N and P35S:AtCGL160C Imaging-PAM panels) to be consistent with the loading scheme of the Northern blot analysis. Size markers are now indicated.

3) Supplemental Figure 3: This figure is somewhat confusing to me. The upper panels are the first exposure to the AtCGL160N antibody, but the upper panel left shows the presence of CF1 signals. In the bottom of the CBB-stained membranes, I wonder if the antibody used for the left panel is the anti-AtCGL160 antibody.

And in the text, the authors describe in L161 that AtCGL160N could not be detected in either stromal or thylakoid extracts (Supplemental Figure 3), but only the proteins in the thylakoid membranes are shown.

Response: We agree with the reviewer that Supplemental Fig. 3 is confusing. Therefore, we split Supplemental Fig. 3 into panel A and B. The Figure legend was rephrased accordingly and should be clearer now.

We added results in a new Supplemental Figure (Supplemental Fig. 3C in the new manuscript) which shows AtCGL160/AtCGL160N immunodetection in protein extracts isolated from rosette leaves. AtCGL160N could not be detected in P35S:AtCGL160N leaf extract. We corrected the corresponding passage in the main text ['P35S:AtCGL160N failed to complement the mutant phenotype (Fig. 2B, C, Supplemental Fig. 2A) and AtCGL160N could not be detected in either thylakoid (Supplemental Fig. 3B) or leaf extracts (Supplemental Fig. 3C).']

<u>Reviewer #3:</u>

This manuscript by Reiter et al clarifies to role of the AtCGL160 protein in Arabidopsis thaliana, which has been shown in previous work to be involved in the assembly of the chloroplast ATP synthase. The current work identifies a specific role for the N-terminal domain of AtCGL160 in late recruitment of CF1 subunits. Using nicely designed protein-protein interaction assays, the authors demonstrate that this likely occurs via interactions between specific regions of AtCGL160N and CF1-β. By heterologously expressing the Synechocystis sp PCC 6803 orthologue of AtCGL160 in Arabidopsis, and observing partial recovery of phenotype, the authors also suggest that the function of the protein has been evolutionarily conserved from cyanobacteria to seed plants.

The work is generally of very high quality, the experiments have been well designed and analysis has been carried out meticulously. Findings are of great interest to the community. However, I have several concerns that should be addressed before publication.

Major Revisions (in prioritized order):

1) In order to assess the significance of the results from the PpsbA2 :synatp1 Synechocystis strain, it would be vital to know how the Atp1 protein content compares to WT levels under the native promoter. Authors assume it is higher,

which quite likely could be the case, but data on protein or at least transcript levels would be required support the conclusion that higher expression level of SynAtp1 doesn't affect growth, O_2 exchange, or ATP synthase assembly.

Response: We now present data on synatp1 expression in a new Supplemental Figure (Supplemental Fig. 9C in the revised manuscript) and describe them in the result section. Quantitative real-time PCR analyses revealed that synatp1 expression levels in PpsbA2:synatp1 and Ppsb A2:Δsynatp1 was increased ~4-fold and decreased to ~10%, respectively, compared with expression levels of the wild-type control.

2) Abstract: There is in fact no direct data showing that photosynthesis is impaired in plants lacking AtCGL160N specifically. Figure 8C shows lower PSII quantum yield for the atcgl160 knockout but no relevant data on 35S:AtCGL160C is shown. Authors should either add relevant data or modify the abstract.

...and related to the above, line 254: I don't think the elevated pmf in AtCGL160C-overexpressing plants alone (Fig. 4B) can be used to conclude downregulation of photosynthesis. Yes, it will likely lead to increased photosynthetic control of PQH2 oxidation at the cyt b6f complex, but no data in Fig. 4 shows that. Perhaps consider showing data for the proton flux parameter vH+, which may be lower in the OE line despite high pmf in comparison to WT, due to low conductivity.

Response: We agree that direct evidence for impaired photosynthesis in P35S:AtCGL160C was missing. We present now proton flux parameters for all genotypes in Fig. 4D and determined lower vH+ values for P35 S:AtCGL160C plants compared to the wild-type control. We also provide Y(II) and NPQ imaging PAM data at 185 µmol photons m-2 s-1 recorded during a light saturation curve (LSC) analysis (Fig. 4A). At this light intensity, the differences in PSII quantum yields were the most obvious and lowered PSII quantum yields for P35S:AtCGL160C compared to the wildtype could be detected. NPQ values at 185 µmol photons m-2 s-1 are now shown and earlier NPQ data were replaced. Since the effect of the missing N-terminal domain of AtCGL160 on photosynthesis could be demonstrated, we did not change the abstract and concluded that photosynthesis is downregulated in P35S:AtCGL160C.

3) The title of the manuscript concise and to the point, but does quite little to distinguish the current work from previous publications on AtCGL160, most importantly Rühle et al. 2014 and Fristedt et al. 2015. I suggest modifying the title to better emphasize the novel findings of the current work.

Response: We replaced the title: 'CGL160-mediated recruitment of coupling factor 1 is required for efficient thylakoid ATP synthase assembly, photosynthesis and chloroplast development in Arabidopsis'

Minor revisions:

1) 199-201: These conclusions appear reasonable based on the electron micrographs, but would benefit from simple quantifications.

Response: We now provide quantification of grana number per chloroplast and grana height in a new Supplemental Figure (Supplemental Fig. 4 in the revised version of the manuscript).

2) Figure 4 legend: gH+ is calculated as the inverse of the time constant, not the constant itself. ...and related, lines 722-726: Description of gH+ measurement is a bit unclear. I assume the authors mean that DIRK kinetics were observed after 2 s illumination, followed by 30 s darkness? And this cycle was repeated 5 times to average the DIRK traces? I also wonder how much the repeating cycles with 30 s dark in between affects gH+, is it necessary to average several DIRKs per replicate? And again, gH+ is the reciprocal (or inverse) value of the time constant from the first order fit to ECS decay kinetics. This would be the same as "lifetime of the decay", but the "inverse of the lifetime" (as used in Materials and Methods) seems confusing to me.

Response: We corrected the paragraph in the Figure legend: 'Proton conductivity of the thylakoid membrane was determined from ECS signal relaxation rates, which were fitted to a first-order decay function. The inverse of the calculated rate constant was expressed as gH+ [s-1]'.

The method part for the determination of gH+ was incorrectly described. We now write: 'For proton conductivity, electrochromic band-shift kinetics were recorded in the millisecond range in 5 consecutive periods of darkness (2 s), separated by light intervals of 30 s, respectively. Averaged signals were fitted to a single exponential decay function and the reciprocal value of the ECS decay time constant was used to estimate the proton conductivity gH+…'

The DIRK protocol and averaging of the fast P515 kinetics to reduce the noise amplitude and to improve fitting were described in Schreiber and Klughammer (2008, New accessory for the Dual-PAM-100: The P515 / 535 module and examples of its application. PAM Appl. Notes 10: 1–10.). We added the reference in the manuscript.

3) It should be pointed out that a very high level of the chimeric AtCGL160N-SynATP1 protein was required to achieve partial restoration in CF_1-CF_0 content in Arabidopsis. While this does show conservation of function at least to some extent, the potential effect of the overexpression should be made clear. It would be interesting to also see ECS data from the plants expressing the chimeric protein, in particular whether the cyanobacterial assembly factor affects the conductivity (gH^+ parameter) or other properties of the ATP synthase.

Response: ECS (pmf, gH+ and vH+) data are now shown for P35S:AtCGL160N-Synatp1 overexpressor plants (Fig. 8E, F, G). Pmf and gH+ were restored and vH+ remained unaltered in P35S:AtCGL160N-SynATP1 overexpressor plants.

We write now in the result section:'…In summary, SynAtp1 could substitute AtCGL160C and function in the chloroplast CF1-CFO assembly process. However, a very high level of the chimeric AtCGL160N-SynAtp1 protein was required to achieve partial restoration in CF1-CFO content in Arabidopsis.…'

4) Figure 7 nicely shows how Y2H was used to map the interaction site between AtCGL160N and CF_1-β, but the figure legend is lacking information about replicates for the Y2H experiments.

Response: Information about replicates of the Y2H experiments are now indicated. We carried out experiments at least twice in independent co-transformation assays.

5) Line 709: Please indicate how the NPQ parameter was calculated.

Response: We now specify in the methods section how the NPQ parameter was calculated [NPQ=(Fm-Fm')/Fm'].

Additional notes from the authors

1) The annotations of the BD-AtCGL160N Y2H constructs Δ29-74 aa and Δ75-105 aa (Figure 7C) were not correct and are now renamed to Δ29-73 aa and Δ74-105 aa in the revised manuscript. We apologize for the error.

2) Spelling error in the legend of Figure 3: It should be 'atcgl160-1' and not 'atclg160- 1'.

3) Spelling error in the main text concerning the results of the co-IP: 'As expected, AtCGL160 was pulled down efficiently from P35S:AtCGL160 extracts (log2 FC ~6.5)'. In the old version, we wrote mistakenly '… from P35S:AtCGL160C extracts…'.

TPC2020-RA-00301R1 2nd Editorial decision – *acceptance pending* Sept. 25, 2022

We are pleased to inform you that your paper entitled "CGL160-mediated recruitment of coupling factor 1 is required for efficient thylakoid ATP synthase assembly, photosynthesis and chloroplast development in Arabidopsis" has been accepted for publication in The Plant Cell, pending a final editorial review by a science editor.

Final acceptance from Science Editor Oct. 11, 2022
